# Advances in Emerging Photonic Memristive and Memristive‐Like Devices

**DOI:** 10.1002/advs.202105577

**Published:** 2022-08-09

**Authors:** Wenxiao Wang, Song Gao, Yaqi Wang, Yang Li, Wenjing Yue, Hongsen Niu, Feifei Yin, Yunjian Guo, Guozhen Shen

**Affiliations:** ^1^ School of Information Science and Engineering Shandong Provincial Key Laboratory of Network Based Intelligent Computing University of Jinan Jinan 250022 China; ^2^ School of Integrated Circuits and Electronics Beijing Institute of Technology Beijing 100081 China

**Keywords:** artificial visual system, brain‐like computing, memristive‐like behavior, memristors, photoelectric logic, photonic memristive devices, resistive switching

## Abstract

Possessing the merits of high efficiency, low consumption, and versatility, emerging photonic memristive and memristive‐like devices exhibit an attractive future in constructing novel neuromorphic computing and miniaturized bionic electronic system. Recently, the potential of various emerging materials and structures for photonic memristive and memristive‐like devices has attracted tremendous research efforts, generating various novel theories, mechanisms, and applications. Limited by the ambiguity of the mechanism and the reliability of the material, the development and commercialization of such devices are still rare and in their infancy. Therefore, a detailed and systematic review of photonic memristive and memristive‐like devices is needed to further promote its development. In this review, the resistive switching mechanisms of photonic memristive and memristive‐like devices are first elaborated. Then, a systematic investigation of the active materials, which induce a pivotal influence in the overall performance of photonic memristive and memristive‐like devices, is highlighted and evaluated in various indicators. Finally, the recent advanced applications are summarized and discussed. In a word, it is believed that this review provides an extensive impact on many fields of photonic memristive and memristive‐like devices, and lay a foundation for academic research and commercial applications.

## Graphical abstract

With the unique characteristic, photonic memristive and memristive‐like devices significantly facilitate the construction of neuromorphic computing and artificial visual systems. This review elaborates on the resistive switching mechanisms, active layer materials, and potential applications in the scientific and industrial of photonic memristive and memristive‐like devices, aiming to provide a systematic and comprehensive insight into its development.

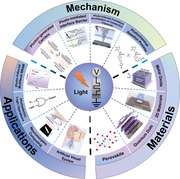

## 1 Introduction

Being the fourth passive device besides the resistor, capacitor, and inductor, the memristor also called memristive device has shown great potential in constructing non‐volatile memory devices,^[^ ^1^, ^2^, ^3^, ^4^, ^5^, ^6^ ^]^ intelligent logic computing,^[^ ^7^, ^8^, ^9^, ^10^, ^11^, ^12^ ^]^ and neuromorphic computing^[^ ^13^, ^14^, ^15^, ^16^, ^17^, ^18^ ^]^ since it was proposed by Leon O. Chua in 1971.^[^ ^19^ ^]^ A memristor is a kind of charge‐controlled or flux‐controlled electronic device, and it possesses a distinctive “fingerprint” characterized by a pinched hysteresis loop.^[^ ^20^, ^21^, ^22^ ^]^ With the advancement of theory and technology, various memristive devices have been proposed and their excellent electrical properties are demonstrated. However, its intrinsic problems, such as instability and high randomness, remain unresolved. In recent years, many emerging approaches, such as exploiting the phase transition properties of materials and the charge trapping behavior of flash memory, have been implemented to optimize these issues and broaden their application scenarios.^[^ ^23^, ^24^, ^25^, ^26^ ^]^ Whether these unique devices can be classified as memristors is debatable at this stage even though they exhibit hysteresis curve behavior similar to memristors.^[^ ^27^, ^28^, ^29^ ^]^ In order to avoid confusion, the name “memristive‐like devices” is preferred to use (memristive devices and memristive‐like devices are strictly distinguished in this review). Until now, memristive and memristive‐like devices have set off a huge research interest, and they prove to be the indispensable elements in the next generation of computer architecture owing to their small size, simple structure, fast response, low power, and the potential to eliminate the von Neumann bottleneck.^[^ ^30^, ^31^, ^32^ ^]^ As the main stimulus to operate the memristors, the electric field is the broadest approach to regulate the memristor transitions between different states.^[^ ^16^ ^]^ However, the researchers have found that the electric field has obvious obstacles, such as single function, weakness expansion, and device vulnerability, even though it is facile to control.^[^ ^33^, ^34^, ^35^, ^36^, ^37^, ^38^ ^]^ To extend the application of the memristors, additional external stimuli are introduced, such as magnetic field,^[^ ^39^, ^40^, ^41^, ^42^, ^43^ ^]^ temperature,^[^ ^44^, ^45^ ^]^ external pressure,^[^ ^46^ ^]^ etc. In particular, these stimuli are orthogonal with the electric field when operating the memristor. Specifically speaking, the resistance states of the memristor can be independently regulated by the electric field or magnetic field, while new resistance states emerge the moment the two parameters act together. Although tremendous external stimuli have been proposed to expand the applicability of the memristor and improve its performance, the un‐stability, high crosstalk, and uncontrollability of these stimuli lead to limitations in practical application. Given the above, the optimal external stimuli to improve the operation efficiency of the memristor is urgently needed.

As a common external stimulus, light has evident merits in various application scenarios after coupling with the electric field due to the controllable, non‐contact, non‐destructive, and low power consumption properties.^[^ ^47^, ^48^, ^49^, ^50^, ^51^, ^52^, ^53^, ^54^, ^55^ ^]^ For instance, based on the electrical‐induced resistive switching, several ON/OFF ratios with distinct differences can be obtained by varying light wavelengths and intensity,^[^ ^49^, ^56^, ^57^ ^]^ which motivates the research and development of light‐induced multilevel resistance random access memory (RRAM) due to the capability of improving the storage density and reducing the size and power consumption of the chip. Furthermore, because of the non‐volatility of the light‐induced resistive switching behavior, some researchers apply the photonic memristive device to the Boolean logic operation that includes “AND,” “OR,” “NOT,” and “XOR.”^[^ ^58^, ^59^ ^]^ Based on this, the optoelectronic reconfigurable logic operations are realized by designing the hardware circuit.^[^ ^7^ ^]^ The concept with respect to the integration of storage and calculation paves a way to break the bottleneck of the traditional Von Neumann computer architecture and provides a practical implementation scheme for the novel computer architecture system in the future.^[^ ^60^ ^]^ In addition, it is well known that the human nervous system works as a core of highly efficient and low‐power parallel operation and is composed of 10^11^ neurons connected by 10^15^ synapses.^[^ ^48^, ^61^ ^]^ As the optimal electronic device in constructing biological artificial synapses, the memristive devices have similar information transmission and processing capabilities with the human brain, as well as constructing artificial neural networks to realize neuron morphological computing.^[^ ^62^, ^63^, ^64^ ^]^ Unfortunately, vision, as the main method of human perception of the world, cannot be imitated by pure electronic devices.^[^ ^65^ ^]^ Vision is the subjective sensation that the human body's photosensitive system feels the light of a specific wavelength from the outside, of which core lies in capturing the light information of the outside world. Inspired by the biological vision, the researchers introduce the external light based on pure electronic synapses and utilize the properties of photosensitive materials which can capture the external light information to realize the photonic synapses.^[^ ^65^, ^66^, ^67^ ^]^ Under light illumination and an electric field, photonic synapses can perform biological synaptic functions such as excitatory post‐synaptic current (EPSC), spike‐timing‐dependent plasticity (STDP), spike‐rate‐dependent plasticity (SRDP), short‐term memory (STM)/long‐term memory (LTM), and paired‐pulse facilitation/depression (PPD).^[^ ^68^, ^69^, ^70^, ^71^, ^72^, ^73^, ^74^ ^]^ Benefiting from the sensitivity to light, the photonic synapses can effectively capture the external optical signals, then eventually convert them into electrical signals to achieve the function of simulating the human visual neural network, which provides a breakthrough for the future memristive devices in the biomimetic synapse and visual neural network. With the rapid development of photonic memristive devices and memristive‐like devices, a large number of researchers are devoted to the research of high‐performance, multifunctional memristors. However, the mechanisms, materials, and specific applications of the photonic memristives and memristive‐like devices have not been categorized and summarized in detail until now.^[^ ^75^, ^76^, ^77^ ^]^

**Figure 1:**
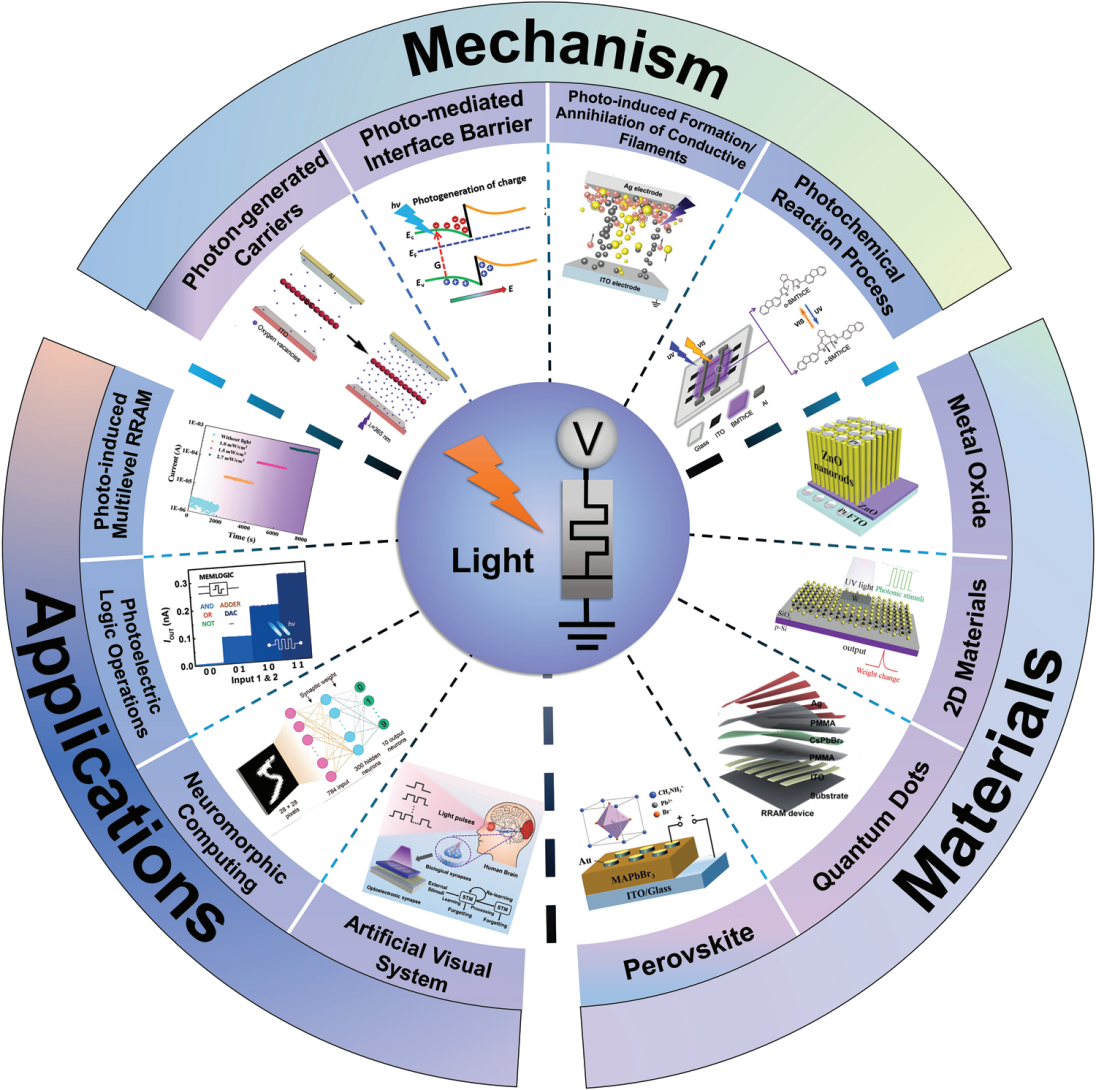
Schematic diagram of the mechanisms, materials, and applications of the photonic memristive and memristive‐like devices. Image for “photo‐generated carriers”: Reproduced with permission.^[^^]^ Copyright 2019, Elsevier B.V. Image for “Photo‐mediated interface barrier”: Reproduced with permission.^[^ ^101^ ^]^ Copyright 2018, American Chemical Society. Image for “Photo‐induced Formation/annihilation of Conductive Filaments”: Reproduced with permission.^[^ ^90^ ^]^ Copyright 2018, Wiley‐VCH. Image for “Photochemical Reaction Process”: Reproduced with permission.^[^ ^126^ ^]^ Copyright 2017, Wiley‐VCH. Image for “Metal Oxide”: Reproduced with permission.^[^ ^145^ ^]^ Copyright 2018, American Institute of Physics. Image for “2D Materials”: Reproduced with permission.^[^ ^99^ ^]^ Copyright 2018, Wiley‐VCH. Image for “Quantum Dots”: Reproduced with permission.^[^ ^90^ ^]^ Copyright 2018, Wiley‐VCH. Image for “Perovskite”: Reproduced with permission.^[^ ^100^ ^]^ Copyright 2018, Wiley‐VCH. Image for “Photo‐induced Multilevel RRAM”: Reproduced with permission.^[^ ^258^ ^]^ Copyright 2021, Wiley‐VCH. Image for “Photoelectric Logic Operations”: Reproduced with permission.^[^ ^7^ ^]^ Copyright 2017, American Chemical Society. Image for “Neuromorphic Computing”: Reproduced with permission.^[^ ^292^ ^]^ Copyright 2021, American Chemical Society. Image for “Artificial Visual System”: Reproduced with permission.^[^ ^72^ ^]^ Copyright 2021, Wiley‐VCH.

In this progress report, we focus on providing a review of emerging photonic memristive and memristive‐like devices based on the recent advanced reports from the view of mechanisms, materials, and specific applications (**Figure** **1**). In detail, we firstly introduce the resistive switching mechanisms of the photonic memristive and memristive‐like devices that are classified into photo‐generated carriers, photo‐mediated Schottky barrier, photo‐induced formation/annihilation of conductive filaments, photo‐induced phase transition, photo‐induced chemical reaction/conformation change, and other mechanisms. Additionally, on the basis of the mechanisms, the classification of the materials that constitute the photonic memristive and memristive‐like devices is discussed in‐depth, which is roughly divided into metal oxides, perovskites, quantum dots, up‐conversion particles, biomaterials, ferroelectric material, chalcogenide compounds, and optical polymers. The characteristics and superiorities of various materials in constructing photonic memristive and memristive‐like devices are deeply studied. Afterward, the specific application scenarios of photonic memristive and memristive‐like devices are elaborated. Finally, the application potential in next‐generation memory systems, logic computing, and the construction of artificial neural networks are outlined, and a clear mini‐roadmap is provided to guide researchers’ efforts. In short, this review summarizes the recent advanced research progress on emerging photonic memristive and memristive‐like devices and guides its future development direction.

## 2 Photo‐Induced Resistive Switching Mechanism

Since the effect of light on memristive devices is based on electrical regulation, detailed elaboration of the electrical regulation mechanism is essential. Typically, traditional resistive switching mechanisms can be divided into three types, including the interface‐type, filamentary‐type, and the pure electron conduction‐type. Interface‐type is one of the important mechanisms in traditional memristors, which refers to the contact barrier between two materials (electrode/active layer or active layer/active layer) that can be tuned by the external electric field, resulting in the conductance transition of the memristors to realize the resistive switching behaviors.^[^ ^78^, ^79^, ^80^, ^81^, ^82^ ^]^ The memristors based on the interface‐type mechanism are widely applied in artificial synapses due to the characteristic of gradual conductance. As the most used mechanism, filamentary‐type is different from the interface‐type, which can achieve the transformations between high resistance state (HRS) and low resistance state (LRS) by forming the conductive filaments in the active layer under the electric field.^[^ ^83^, ^84^, ^85^ ^]^ Furthermore, the filamentary‐type can be detailed divided into electrochemical metallization (ECM) and valance change mechanism (VCM) according to the type of ions that compose the conductive filaments (oxygen vacancies, metal ions, rare earth elements, etc.).^[^ ^86^ ^]^ Especially, the transformation from LRS (HRS) to HRS (LRS) is abrupt, so most of them are used in the resistive storage and logic calculation.^[^ ^52^, ^53^ ^]^ Different from the above two kinds of ionic conduction‐based mechanisms, the conductive carriers of pure electron conduction‐type are electrons‐based and generally induced by the capture of electrons by the inherent defects of the material.^[^ ^87^, ^88^ ^]^ The pure electron conduction‐type based memristive devices have exhibited great potential in constructing flexible transparent memristors because it exists mostly in organic–inorganic hybrid memristive devices. The above‐mentioned interface barriers, conductive filaments, and traps mentioned also perform a role in the mechanism of photonic memristive and memristive‐like devices.^[^ ^7^, ^89^, ^90^ ^]^ Here, we discuss the process of light acting on photonic memristive and memristive‐like devices in detail according to the mechanism of conventional memristors.

### 2.1 Photo‐Generated Carriers

**Figure 2:**
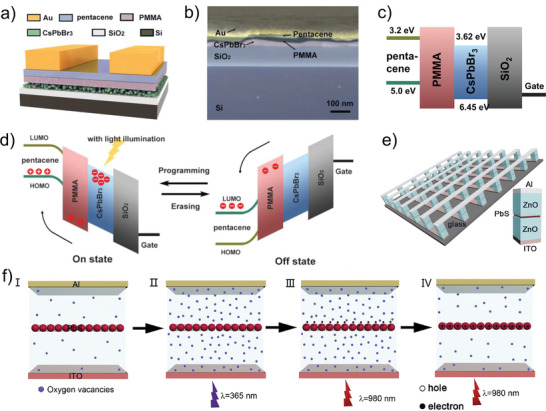
a) 3D schematic illustration of the CsPbBr_3_ QDs‐based photonic memristive‐like device. b) Side view of the Au/pentacene/PMMA/CsPbBr_3_/SiO_2_/Si device recorded by the cross‐sectional SEM. c) Schematic energy diagram of the device at initial state. d) Schematic energy diagram of the device during a light programming operation and electrical erasing operation under dark conditions. e) 3D schematic of the Al/ZnO/PbS QDs/ZnO/ITP structure and the conceptual drawing of the device array. f) Resistive modulation mechanisms of the Al/ZnO/PbS QDs/ZnO/ITO device in the full‐photo mode. (a–d) Reproduced with permission.^[^ ^98^ ^]^ Copyright 2018, Wiley‐VCH. (e, f) Reproduced with permission.^[^ ^64^ ^]^ Copyright 2019, Elsevier B.V.

The capacity of photon information detecting and photon energy collection in the photoelectric sensors and solar cells can be attributed to the emergence of built‐in potential caused by the excitation of photo‐generated carriers inside the materials under the illumination condition.^[^ ^91^, ^92^, ^93^, ^94^ ^]^ Photo‐generated carriers significantly increase the current flowing through the device under light, which realizes the characteristics of the photo‐modulated conductance. Inspired by controllable modulation of the photocurrent, the effect of photo‐generated carriers is applied to the memristive devices to realize the light‐induced resistive switching behaviors. This controllability is embodied in the memristive devices, where the resistive state of the memristive devices can be switched by modulation of voltage or removal of light. Typically, a photo‐programming‐electric‐erased photonic memristive‐like device based on Au/pentacene/poly(methyl methacrylate)(PMMA)/CsPbBr_3_ quantum dots (QDs)/SiO_2_/Si is proposed by Han et al.^[^ ^95^ ^]^ The device diagram and scanning electron microscope (SEM) image are exhibited in **Figure** **2**a,b, respectively, in which the pentacene layer serves as the conductive channel while the CsPbBr_3_ QDs act as the active layer. The energy band arrangement diagram of the proposed devices is shown in Figure 2c. During the process of photo‐programming, a large number of free‐moving photo‐generated electron–hole pairs can generate inside the CsPbBr_3_ QDs layer under white light irradiation, while the photo‐generated holes are transferred from the CsPbBr_3_ QDs to the pentacene layer through band bending, leaving the photo‐generated electrons in the conduction band of CsPbBr_3_. Furthermore, the effect of holes swept into the semiconductor channel is accelerated owing to the enhancement of the additional internal electric field by trapped electrons. After removing the light, the holes in the semiconductor channel can be retained by the potential well for a long time, resulting in an increase in the device conductance and enabling the photo‐programming process. Subsequently, as the electrons in the CsPbBr_3_ QDs layer are removed by the external electric field, the device conductance drops, and the electrical erasing process is realized (Figure 2d). It should be mentioned that the process of the device returning to the initial HRS is realized by applying negative voltage instead of illumination. To further simplify the operation and reduce the power consumption, this group proposes an photonic memristive device based on the ITO/ZnO/PbS QDs/ZnO/Al (Figure 2e), which can realize the light‐induced enhanced and suppressed conductance under various wavelengths of light (full‐photo modulation).^[^ ^64^ ^]^ The explanation of the resistive switching mechanism is shown in Figure 2f. In the dark condition, the concentration of oxygen vacancies in the ZnO layer is low (corresponding to process I in Figure 2f). When the ultraviolet (UV) light is turned on, the additional carriers and oxygen vacancies can be excited due to the photo‐sensitiveness of ZnO, leading to the gradual enhancement of the device conductance (corresponding to process II in Figure 2f). As the time of UV radiation increases, the accumulated oxygen vacancies lead to the synaptic excitatory post‐potential, realizing the enhancement of synaptic weight. The electrons can also be excited in the PbS QDs under infrared (NIR)‐light, which are immediately trapped by oxygen vacancies in the ZnO to change them from carrying two positive charges to neutral particles. Different from the promotion of device conductance under UV light, the trapped electrons by oxygen vacancies under NIR‐light significantly reduce the conductivity of the device, resulting in the depression of the synaptic weight (corresponding to processes III and IV in Figure 2f). The full‐photo regulation characteristics demonstrate that photo‐generated carriers can produce varied responses to conductance under different designs, enabling photonic memristive devices to be used for a considerably wide variety of applications. Until now, the exploration of photo‐generated carriers is continuing, especially to effectively control the direction of their migration or diffusion to improve the stability of photonic memristive device.^[^ ^65^, ^96^, ^97^, ^98^ ^]^

### 2.2 Photo‐Mediated Interface Barrier

**Figure 3:**
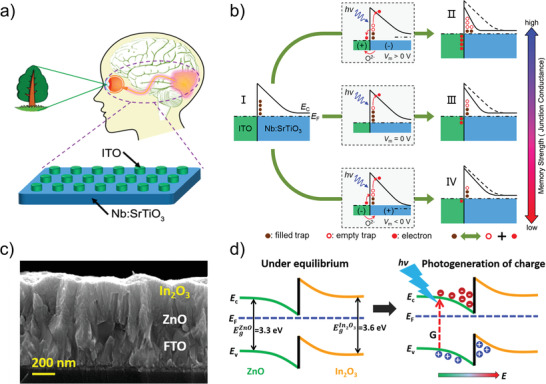
a) Conceptual drawing of the ITO/Nb: SrTiO_3_ heterojunction artificial photonic synapse. b) Resistive switching mechanism of the ITO/Nb: SrTiO_3_ photonic synapse. I) Initial Schottky barrier profile. II) Schottky barrier profile after light illumination accompanied by positive voltage stress. III) Schottky barrier profile after only light illumination. IV) Schottky barrier profile after light illumination accompanied by negative voltage stress. The dashed lines in (II–IV) represent the initial energy band profile. c) Cross‐sectional SEM image of the In_2_O_3_/ZnO/FTO device. d) Band alignment at the ZnO/In_2_O_3_ interface under thermal equilibrium and photo‐induced electron‐hole pair generation and their respective trapping on the ZnO and In_2_O_3_ sides. (a, b) Reproduced with permission.^[^ ^65^ ^]^ Copyright 2019, American Chemical Society. (c, d) Reproduced with permission.^[^ ^101^ ^]^ Copyright 2018, American Chemical Society.

As a kind of external stimuli source, light can effectively excite the electron–hole pairs and alter the concentration of the carriers at the interface when irradiating the memristors, finally affecting the contact barrier. For memristors with contact interfaces, the control of their photoresponse is achieved by tuning the contact barriers between various materials.^[^ ^94^, ^95^, ^96^, ^97^, ^98^, ^99^, ^100^, ^101^ ^]^ Among them, the Schottky barrier formed at the semiconductor‐metal interface is the most typical mechanism in the type of photo‐mediated interface barrier. Based on the modulation of the Schottky barrier at the interface, Li et al. construct the photonic synapses with ITO/Nb: SrTiO_3_ structure (**Figure** **3**a).^[^ ^65^ ^]^ The gradual regulation of the conductance of the photonic synapses is achieved by a mixed spike consisting of voltage and light stimulation, of which detailed processes are shown in Figure 3b. Thereinto, the electron affinity energy of the Nb: SrTiO_3_ is ≈3.9 eV and the work function of the ITO is ≈4.5 eV. The unmatched energy levels lead to the formation of the Schottky barrier between the electrode and the active layer, which is illustrated in Figure 3b–I. The transport of the electrons at the ITO/Nb:SrTiO_3_ interface is hindered by the height/width Schottky barrier, resulting in the HRS of the Nb:SrTiO_3_ heterojunction photonic synapse in the initial state. Under the effect of light, electrons trapped by oxygen vacancy defects inside the Nb:SrTiO_3_ material are released into the electrode (from Nb:SrTiO_3_ to ITO), and, leaving positive charged empty traps capable of reducing the built‐in electric field, leading to the reduction of the Schottky barrier at the heterojunction interface. Finally, the resistance of the Nb:SrTiO_3_ heterojunction photonic synapse is decreasing with the Schottky barrier, as shown in Figure 3b‐III. When the positive voltage is applied at the ITO electrode (*V*_m_ > 0), the electrons are migrated from the Nb:SrTiO_3_ to the ITO under the electric field, leaving oxygen vacancies in the Nb:SrTiO_3_. After applying the illumination to the devices, many photo‐generated electrons are released into the Nb:SrTiO_3_, while the separation of electrons and oxygen vacancies is promoted by the electric field, which is illustrated in Figure 3b‐II. The interaction between the electric field and light reduces the height and width of the Schottky barrier at the interface, leading to the lower resistance of the Nb:SrTiO_3_ heterojunction photonic synapse. On the contrary, when the negative voltage is applied at the ITO electrode (*V*_m_ < 0), the resistivity of the Nb:SrTiO_3_ heterojunction photonic synapse is enhanced due to the suppressed photo‐response intensity, as shown in Figure 3b‐IV. The modulation of the Schottky barrier at the electrode/active interface is mainly achieved by the response of the defect in the material of the resistive layer to light. Light can tune the contact barrier at the semiconductor/semiconductor heterojunction interface in addition to the effect on the Schottky barrier at the metal/semiconductor interface. Kim et al. construct the FTO/ZnO/In_2_O_3_ photonic synapses based on type‐II heterojunction, of which cross‐sectional SEM image is shown in Figure 3c.^[^ ^101^ ^]^ The difference in electron affinity between ZnO (4.35 eV) and In_2_O_3_ (3.5 eV) leads to forming of the potential barrier at the contact surface. When the light strikes the photonic synapses, the photo‐generated electron–hole pairs at the ZnO/In_2_O_3_ interface are separated, while the photo‐generated holes are migrated to the In_2_O_3_ and the photo‐generated electrons are migrated to the ZnO under the electric field, as shown in Figure 3d. Finally, the height/width contact barrier at the ZnO/In_2_O_3_ interface is reduced owing to the carrier transport behaviors, which effectively enhances the tunneling probability of electrons and the conductivity of the photonic synapses. In addition, the type‐II heterojunction can also be formed at the 2D material contact interface. An interface barrier‐based memristor distinguished from the above structures is proposed by Guo et al., which is structured by W/MoS_2_/SiO_2_/P‐Si.^[^ ^99^ ^]^ It is pointed out that the conductivity of devices based on the mechanism of the photo‐mediated interface barrier is gradual, which can be ascribed to the gradual variety of the contact barrier under the strike of electric field and light.

### 2.3 Photo‐Induced Formation/Annihilation of Conductive Filaments

**Figure 4:**
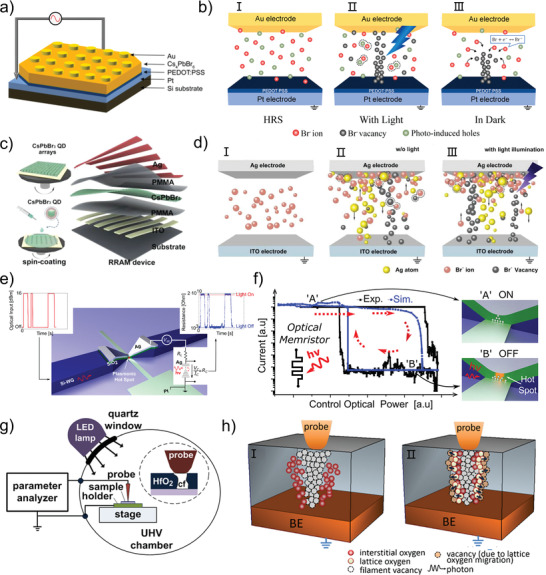
a) Schematic diagram of the Cs_4_PbBr_6_‐based photonic memristive device. b) Schematic diagram of the proposed mechanism for the operation of the Cs_4_PbBr_6_‐based photonic memristive device. I) in the initial state, II) with light and III) in dark. c) Schematic illustration of the CsPbBr_3_ QD‐based photonic RRAM device. d) Illustration of resistive switching at the I) initial state, II) during the set process under dark conditions, III) and during the set process under UV illumination. e) Schematic diagram of the ultra‐scaled memristive photodetector. A modulated input optical power (left panel) is converted into a digital electronic current (right panel). f) Memristive current as a function of the optical input power (black and red solid circles are experiments, and blue circles represent simulations), and the ON‐state (OFF‐state) where the filament is completely formed (dissociated). g) Schematic of the experimental setup used in this study and the inset show the structure of the HfO_2_‐based RRAM. h) Schematic illustration of the proposed mechanism for the light‐induced resistance reset. I) Formation of a conducting filament comprising oxygen vacancies, with the released oxygen ions populating interstitial sites in the filament vicinity. II) Partial filament disruption due to limited supply of photo‐excited migrating oxygen ions, including weakly bonded lattice oxygen in the filament vicinity. (a, b) Reproduced with permission.^[^ ^105^ ^]^ Copyright 2019, Elsevier B.V. (c, d) Reproduced with permission.^[^ ^90^ ^]^ Copyright 2018, Wiley‐VCH. (e, f) Reproduced with permission.^[^ ^110^ ^]^ Copyright 2018, American Chemical Society. (g, h) Reproduced with permission.^[^ ^111^ ^]^ Copyright 2019, American Institute of Physics.

The conductive filament mechanism, which is one of the most typical mechanisms applied in traditional memristive devices, exhibits a gratifying effect in emerging photonic memristive and memristive‐like devices.^[^ ^102^, ^103^, ^104^ ^]^ There are some differences in the principle of photo‐excitation on the conductive filament of different ion types. In detail, the effect of light on conductive filaments is diverse and depends on the material of the active layer and the electrode. In most cases, light can promote the formation of conductive filaments whether it is VCM or ECM. The particles that make up the conductive filament are valence electrons in VCM, and the most typical effect of light on it is Br^−^. In the Au/Cs_4_PbBr_6_/poly(3,4‐ethylenedioxythiophene)‐poly(styrenesulfonate) (PEDOT:PSS)/Pt structured photonic memristive device (**Figure** **4**a) proposed by Chen et al., the transition between HRS and LRS is based on the photo‐induced formation/rupture of the Br^−^ conductive filament.^[^ ^105^ ^]^ The device exhibits HRS in the initial state, that is, the state in which the conductive filament is not formed, as shown in Figure 4b‐I. When the light reaches the device, the photoinduced holes (h^+^) can be excited in the perovskite‐based materials, then the holes react with Br^−^ ions (Figure 4b‐II). The reaction can be expressed as Br^−^ + H^+^ ↔ Br, indicating an accumulation of Br^−^ ion vacancies with increasing corresponding photocurrent (Figure 4b‐III). Similarly, when active electrode is selected, the ECM‐based conductive filament can be significantly affected. For instance, the Ag/PbS QDs@PMMA/ITO‐based photonic memristive device proposed by Zhang et al. illustrates the role of light in the metal conductive filament.^[^ ^106^ ^]^ Because of the photovoltaic effect, the electron‐hole pairs are excited in the PbS QDs when the photon energy is higher than the energy of the bandgap. Then the electron‐hole pairs are separated at the PbS–PMMA interface and move toward the electrodes under the influence of the electric field, enhancing the redox reaction of the Ag and ITO electrodes. Therefore, it is much easier to form the Ag conductive filament, proving the positive effect on the formation of the Ag conductive filament. Apart from the above phenomenon, the more complicated situation occurs in photonic memristive devices where ECM and VCM coexist. Han et al. have done a series of works in the light act on these devices, including typical research on Ag/PMMA/CsPbBr_3_ QDs/PMMA/ITO based photonic RRAM (a kind of memristive devices) (Figure 4c).^[^ ^90^ ^]^ Under the dark condition, the conductive carriers in the device are Br^−^, which can be observed in Figure 4d‐I. After illumination, the electron–hole pairs are excited and separated under the electric field, producing an additional internal electric field. Then, as shown in Figure 4d‐II,III, the movement of the Br^−^ and the oxidized Ag^+^ are promoted by the internal electric field, and the formation of conductive filaments composed of Br^−^ and Ag^+^ is significantly promoted. Additionally, the influence of light‐induced thermal forces on formed conductive filaments, particularly for dissociating these filaments, is not insignificant or even considerable.^[^ ^107^, ^108^, ^109^ ^]^ To better understand the effect of light‐induced thermal forces, the effect of electrothermal generation is briefly discussed first. When a sweeping voltage is applied to the unipolar resistive switching device at LRS, the current flowing across the conductive channel increases dramatically, causing a considerable quantity of Joule heating. Hence, the temperature in the conductive channel area increased rapidly, and the conductive filaments fused to a high degree, changing the resistance state. Similar to electrothermal reactions, light is a source of radiation that causes a substance to heat up. Recently, an ultra‐scaled memristive photodetector is proposed by Leuthold et al., in which the incident light can induce changes in the conductance of the memristor.^[^ ^110^ ^]^ By tuning the power of the incident light, the memristive photodetector exhibits a pinched hysteresis loop between the optical power and conductance, thus it can be termed a memristive‐like device. The device is made up of a pyramid‐like three‐dimensional (3D) plasmonic tip consisting of Ag‐*α*‐SiO_2_‐Pt and a silicon waveguide, as illustrated in Figure 4e. In the initial state, an offset voltage (*V*_dc_) is applied to the Ag electrode, which generates an atomic size filament, while when the light is turned on, the state transforms to the HRS as well as the filaments are fused, as shown in Figure 4f. During the light exposure, the dynamic behavior of Ag atoms, which form conductive filaments, is dominated by two effects. One impact is accelerated electrochemical processes caused by light‐induced local heating, which results in the dissolution of conductive filaments, and the other is the thermally induced lateral diffusion of Ag ions. The former works in a similar way to the electrothermal effect, whereas the latter depends on an effective diffusive force and a rather weak optical force. After increasing the strength of illumination, the diffusive force dominates the drift of Ag ions, pushing the Ag ions away from the filament until it breaks. After removing the light, Ag ions can relocate under the action of the electric field to form conductive filaments, turning back to the LRS. In addition to the above light‐induced local heating, the impact of light on the oxygen vacancies inside the active layer can potentially cause the conductive filaments to rupture. Ang et al. construct a kind of photonic memristive device which is based on HfO_2_/TiN/Ti/p‐Si (Figure 4g).^[^ ^111^ ^]^ The oxygen vacancy conductive filament is formed under the positive voltage pulse (Figure 4h‐I). Then, during subsequent light exposure, a smaller share of the light‐activated interstitial oxygen ions distributed throughout the dielectric volume may potentially reach the filament, resulting in only partial filament disruption (Figure 4h‐II). In summary, light affects the formation and rupture of different kinds of conductive filament, which is depended on the choice of the material that forms the active layer and the electrode material. Until now, the effect of illumination on memristive and memristive‐like devices performance is still to be further studied.

### 2.4 Photo‐Induced Phase Transition

Many devices can undergo a dramatic change in resistivity between their polycrystalline and amorphous under stimuli, either optically or electrically. Since the phase transition property was discovered by Ovshinsky, it has been widely applied in rewriteable devices, electrically written devices, and especially novel phase‐change memories (PCM).^[^ ^112^, ^113^ ^]^ Thereinto, as a non‐volatile resistive switching memory composed primarily of phase‐change materials, PCM shows multiple resistive switching behaviors, and its ability to “remember” previous excitations also imbues them with memristive‐like functionality.^[^ ^114^ ^]^ As a result, it has already been pointed out that electrical PCM cells are a form of memristor.^[^ ^115^ ^]^ Recently, Wright et al. propose that the phase‐change devices can be an optical analogue of a memristor due to the light‐induced resistance transition.^[^ ^116^ ^]^ As we know, the hysteretic current‐voltage curve, which exhibits a non‐linear relationship between the integrals of current and voltage, is the distinctive feature of electrical memristance.^[^ ^117^, ^118^, ^119^ ^]^ Similarly, the optical reflectivity of the photonic PCM is determined by excitation history, and a distinctive pinched hysteresis loop can be performed between reflected and incident light, which can be referred to as a memristive‐like property. As the core of operations in PCM, understanding the phase‐change relative to the optical properties is important for improving the design of the upcoming photonic devices. In this section, the photo‐induced phase transition of the photonic memristive‐like PCM devices is discussed and explained in detail for improving the design of the upcoming photonic devices.

**Figure 5:**
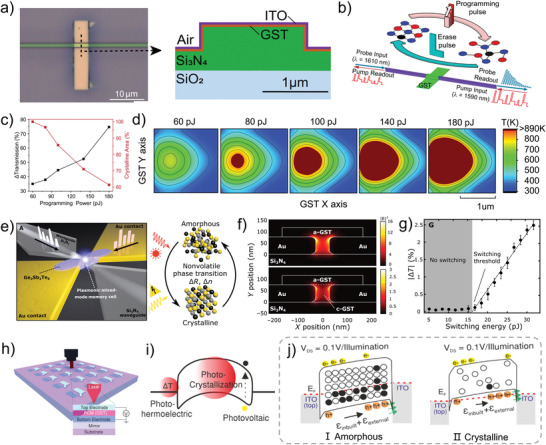
a) Magnified image of GST on top of the waveguide, and schematic cross‐section of the completed device. b) Schematic of the optical pulse shapes used to amorphize and crystallize the integrated PCM cell. c) Simulated transmission and crystalline fraction as a function of the programming pulse energy. d) Simulated temperature distribution in the GST memory cell after a 20 ns programming pulse. e) 3D illustration of the proposed PCM concept. f) Eigenmode simulations of field enhancement inside the plasmonic nanogap when the GST is in the amorphous (top) or crystalline state (the region between Au electrodes, bottom). g) Experimental measurement of total energy in the waveguide required to achieve a nonvolatile phase transition. The switching threshold is measured to be 16 ± 2 pJ according to a linear fit to the data (black dashed line). h) Schematic illustration of the GST‐based device and the measurement setup. i) Concept diagram of the resistive switching mechanism. j) Diagram of electron transport mechanism under Ι) Amorphous and II) Crystalline. (a–d) Reproduced with permission.^[^ ^121^ ^]^ Copyright 2019, Optica Publishing Group. (e–g) Reproduced with permission.^[^ ^122^ ^]^ Copyright 2019, American Association for the Advancement of Science. (h–j) Reproduced with permission.^[^ ^123^ ^]^ Copyright 2018, American Chemical Society.

Typically, the effect of the light on the phase‐change process is simple and clear. The PCM is normally amorphous in the initial state, but it may be crystallized by exposing it to a certain light at a high enough intensity. The exposure light generates thermal energy during this process, which heats the material to a temperature over the glass‐transition temperature, resulting in a phase‐change phenomenon. After that, the material is quenched with a sequence of intense and condensed light to return it to its initial amorphous state. Wall et al. directly measure the change in structure and optical characteristics during the photo‐triggered amorphization of phase‐change material Ge_2_Sb_2_Te_5_ (GST).^[^ ^120^ ^]^ The GST contains abundant resonant bonds in its initial crystalline state, but once it is optically excited at 800 nm, free carriers are generated via interband transitions, leading to the loss of resonant bonding. A non‐equilibrium state of GST is first observed by the femtosecond optical excitation, and then a lattice heating phenomenon is caused by the transfer of the electron energy to the covalent backbone, which finally induces the transform from a non‐equilibrium state into a stable amorphous state. The detailed transition of GST from crystallization to amorphization under photo‐excitation is observed in this study, revealing a new understanding of GST phase transformation. Researchers have previously focused on bipolar resistance state transition of phase‐change materials, that is, crystallization and amorphization, which are inapplicable in the big data era. Bhaskaran et al. demonstrate a precise and repeatable multilevel photonic PCM composed of a GST thin film passivated with ITO and evanescently connected to a Si_3_N_4_ photonic waveguide (**Figure** **5**a) which is programmable using a unique double‐step optical pulse‐modulated technique.^[^ ^121^ ^]^ The fraction of amorphous versus crystalline material of the memory can be controlled by the programming light irradiation through the Si_3_N_4_ photonic waveguide, resulting in changes in the reflectivity and transmittance of the GST thin film. As mentioned above, this property is a kind of memristive‐like functionality because the transmittance of the photonic PCM is determined by excitation history. Figure 5b exhibits the schematic of the phase transition under the optical pulses. It can be observed that the GST undergoes an amorphization process under programming pulses (probe input: 1610 nm and 1590 nm), then, the amorphization is erased by the erase pulses with a trailing rectangular profile, finally returning to the crystalline. During this process, the fraction of amorphous versus crystalline material of the GST can be precisely controlled by varying the programing power, as shown in Figure 5c. The proposed photonic PCM exhibits 34 arbitrary levels, corresponding to over 5 bits, under various degrees of amorphization. Further, a simulation of the temperature distribution at the center of the GST is implemented to verify the programming pulse effect. As shown in Figure 5d, with the increase of the programming pulse, the area of temperature surpasses the melting temperature marked by the dark red gradually increases, indicating that the PCM is gradually amorphized. This work proposes a new modulation strategy for making phase transitions stable and continuous. It should be pointed out that for initiating the phase transition, the ON/OFF ratio between the conductive and insulating state in PCMs demands very close metal contact spacing (usually tens of nanometers), and the total volume of material for light‐matter interaction is limited due to the small conductive region after programming, which reduces the efficiency of device operation. Aiming at these stringent requirements, Bhaskaran et al. combine waveguide‐integrated plasmonic nanogaps with PCMs and presented a GST‐based electro‐optic memory cell with a diffraction limit of tens of nanometers or less, which is significantly lower than that of traditional optical devices.^[^ ^122^ ^]^ The proposed PCM is assembled of a partially etched Si_3_N_4_ rib waveguide, two metal electrodes, and a thin film (75 nm) of GST with a 5‐nm SiO_2_ capping layer, as illustrated in Figure 5e. When optical pulses (7.5 mW for 8 ns followed by 3 mW for 400 ns) are sent through the rib waveguide to the nanogaps, the optical power is absorbed by the GST due to its nonvanishing complex refractive index. The crystalline GST is then melt‐quenched to amorphization, realizing the writing process. A rectangular erase pulses (7.5 mW for 8 ns) are used to crystallize the GST. Using Lumerical Solutions, 2D eigenmode and 3D finite‐difference time‐domain (FDTD) simulations are carried out to quantify the field enhancement of the plasmonic nanogap, as shown in Figure 5f. Due to the confined strong field within the narrow nanogaps, the electric field intensity is enhanced by more than an order of magnitude in the case of amorphous GST. When the GST is erased to the crystalline state, the enhancement reduces by a factor of 5. PCMs and nanoplasmonics work together to drastically minimize metal losses at light frequencies and improve light conduction efficiency.(Figure 5g). The understanding of the underlying mechanism of light‐matter interaction is restricted to photothermal crystallization in the above elaboration, however, it is a multi‐factor interaction that needs to be further explored. As shown in Figure 5h, Bhaskaran et al. provide comprehensive research on the photo‐induced phase transition process, with a proposed device based on the unusual phase‐change material GST.^[^ ^123^ ^]^ When the laser radiates on the device, a strong photoconductive behavior appears, which is induced by a complex interaction of three individual mechanisms: photoconductive, photoinduced crystallization, and photoinduced thermoelectric effects (Figure 5i). During irradiation, photoinduced thermoelectric effects dominate resistive switching behaviors, whereas photoinduced crystallization and photoconductive contributions to the photocurrent are smaller. By tuning the device engineering, such as electrodes and device geometry, the contribution of the photothermal effects to the photocurrent can be effectively controlled. Subsequently, a phase change process occurs under photothermal effects, and in this process, a change of carrier transport behavior during phase transformation is obtained by electrical behavior analysis, that is, from thermionic (Figure 5j‐I) to tunneling (Figure 5j‐II). These results reveal a detailed contribution of light–matter interaction during phase transitions and provide insights into the photophysics of phase‐change materials. At present, the photo‐induced phase transition mechanism has become a reliable and extensive mechanism owing to the improvement of device design and processing technology.

### 2.5 Photochemical Reaction Process

**Figure 6:**
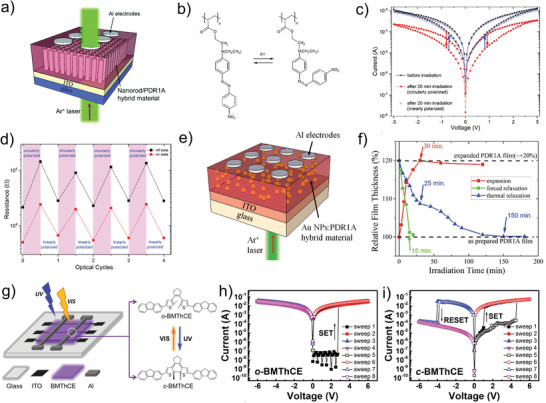
a) Schematic of the photonic memristive devices consisting of PDR1A and b) the transform of PDR1A chromophores chemical structure under circularly polarized light and linearly polarized light. c) Optical modulation of the electronic switching characteristics of a ZnO/PDR1A memristive device before irradiation, directly after irradiation (20 min) with circularly polarized light and linearly polarized light. d) Optical modulation of the HRS and LRS states via repeated irradiation of circularly and linearly polarized light. e) Schematic of the photonic memristive devices consisting of Au nanoparticles (NPs) embedded within a thin film of azobenzene polymer PDR1A and deposited between ITO and Al electrodes. f) Thickness changes of a PDR1A film with time on irradiation with (red line) circularly polarized light of intensity 180 mW cm^−2^. g) Schematic diagram of o‐BMThCE‐based memory, and the chemical structure of the photochromic diarylethene. *I*–*V* characteristics of the BMThCE‐based memories h) ITO/o‐BMThCE/Al, and i) ITO/c‐BMThCE/Al. (a–d) Reproduced with permission.^[^ ^124^ ^]^ Copyright 2017, Royal Society of Chemistry. (e, f) Reproduced with permission.^[^ ^125^ ^]^ Copyright 2019, Wiley‐VCH. (g–i) Reproduced with permission.^[^ ^126^ ^]^ Copyright 2017, Wiley‐VCH.

Unlike the redox reaction of the conductive filaments in traditional memristors, the photochemical reaction process is based on the chemical denaturation of materials caused by light. After denaturation, the resistivity of the material will be significantly different from the initial state and a resistive switching behavior occurs. Kemp et al. propose a photonic memristive device with reversible latched switching that is controllable by light.^[^ ^124^ ^]^ As shown in **Figure** **6**a, the photonic memristive device consists of an optically active azobenzene polymer, poly (disperse red 1 acrylate) (PDR1A), overlaying a forest of vertically aligned ZnO nanorods. Thereinto, PDR1A, as a kind of light‐sensitive material, can undergo a photochemical isomerization upon optical excitation. When irradiated with circularly polarized light, the PDR1A undergoes a *trans‐cis* photochemical isomerization due to the accumulation of the PDR1A chromophores with an out‐of‐plane alignment (Figure 6b), thus leading to the expansion of the polymer PDR1A. After that, the expanded PDR1A increases the contact area between PDR1A and ZnO, which results in the reduction of the device conductance (as shown in Figure 6c). In addition, to switch back the device to its original state, a linearly polarized light is applied to the device (Figure 6d). Different from the circularly polarized light, the PDR1A chromophore tends to in‐plane alignments under the linear polarized light, which leads to the *cis*–*trans* thermal isomerization and the contraction of the PDR1A, and, the device is operated to the original state finally. Similar studies have been reported in Al/Au:PDR1A/ITO structured devices, in which the Au nanoparticles instead of ZnO nanorods are utilized to improve the photo‐response intensity and stability of the device (Figure 6e).^[^ ^125^ ^]^ Figure 6f exhibits the relative thickness of the PDR1A film under various conditions. The observed increase in thickness of the film is ≈20% after the light (red line), and on the removal of the light, the *cis*–*trans* isomerization occurs spontaneously without an electric field (blue line). Under the electric field, the forced relaxation can reset the film to its original thickness in 15 min. Actually, the photochemical reversible reaction is instability and uncontrollable although it can realize the multiple transformations between HRS and LRS. One of the reasons is that the reaction of expansion and contraction is difficult to control, and the other is that the *cis*–*trans* thermal isomerization of PDR1A is incomplete. In a word, the practical applications of the photochemical reversible reaction‐based photonic memristive devices need to be further studied.

Moreover, the photochromophore (BMThCE) with superior stability and endurance under UV‐light has been applied in photonic RRAM by Huang et al.^[^ ^126^ ^]^ As shown in Figure 6g, the BMThCE molecules are in the ring‐open isomers (o‐BMThCE) in the initial state, while it converts to the ring‐closed isomers (c‐BMThCE) after the trigger of UV irradiation. In addition, the BMThCE molecules convert to o‐BMThCE state after applying the visible light (VIS)‐irradiation. In the ring‐open state, the device can only realize the write operation once under the action of the voltage, but cannot achieve the reset operation by applying a negative voltage, as shown in Figure 6h. This is the typical characteristic of write‐once‐read‐many times memory (WORM). After irradiation by UV‐irradiation, the BMThCE‐based RRAM realizes the reset operation under the negative voltage, which indicates that the transform from WORM to the RRAM can be achieved by adjusting the wavelength of light (Figure 6i). Furthermore, compared with PDR1A, the BMThCE shows more stable and controllable properties in terms of resistive switching behaviors.^[^ ^127^, ^128^ ^]^ In fact, there are few reports about photochemical reaction process‐based photonic memristive devices until now which may be due to their great weakness in reversibility, stability, and controllability.^[^ ^129^, ^130^ ^]^

## 3 Active Materials for Photonic Memristive and Memristive‐Like Devices

In recent years, novel memristors with various device structures, application scenarios, and mechanisms have been proposed with the rapid development of big data.^[^ ^131^, ^132^, ^133^, ^134^ ^]^ However, as a kind of light‐sensitive device, selecting the suitable materials that respond to light is fundamental to determining the specific application and mechanism of the device. Various characteristics of the materials applied to photonic memristive and memristive‐like devices should be considered. In detail, the power consumption can be affected by the photo‐response intensity, while the bandwidth and selectivity of light wavelengths are determined by the photo‐sensitive wavelength, and the operating speed of the photonic memristive and memristive‐like devices is dependent on the photo‐response speed. In addition, the stability and reversibility of the device to light are the focus of our research. Based on recent studies, the materials applied to the photonic memristive and memristive‐like devices are summarized. In this section, the advantages and limitations of various kinds of materials applied to photonic memristive and memristive‐like devices are classified and illuminated.

### 3.1 Traditional Metal Oxides

**Figure 7:**
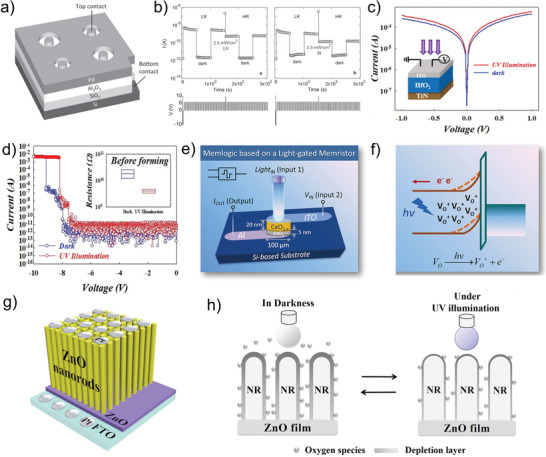
a) Schematic representation of the Pb/Al_2_O_3_/SiO_2_/Si device. b) Data retention capability in dark conditions and under illumination with UV light, and IR light. c) The *I*–*V* characteristics of the ITO/HfO_2_/TiN‐based memristor. Inset: the schematic illustrations of the ITO/HfO_2_/TiN‐based memristor. d) Forming voltage of ITO/HfO_2_/TiN‐based memristor with and without illumination. e) Schematic diagram of the photonic memristive device with ITO/CeO_2−_ *_x_*/AlO*_y_*/Al structure. f) Mechanism for the light‐gate memristive characteristics of the device. g) Schematic illustration of the ZnO nanorods‐based device structure. h) Initial state before UV exposure and after UV exposure of the ZnO nanorods surface. (a, b) Reproduced with permission.^[^ ^137^ ^]^ Copyright 2017, Wiley‐VCH. (c, d) Reproduced with permission.^[^ ^141^ ^]^ Copyright 2020, IOP Science. (e, f) Reproduced with permission.^[^ ^7^ ^]^ Copyright 2017, American Chemical Society. (g, h) Reproduced with permission.^[^ ^145^ ^]^ Copyright 2018, American Institute of Physics.

Metal oxides, as the popular branch of semiconducting material, provide great potential for use in photonic memristive and memristive‐like devices owing to the wide variety, various preparation methods, and excellent compatibility with other materials.^[^ ^135^, ^136^ ^]^ Typically, Mariana et al. propose a Pb/Al_2_O_3_/SiO_2_/Si‐based photonic memristive device, which is the first report that introduces the light signals to the memristor.^[^ ^137^ ^]^ The proposed device structure is shown in **Figure** **7**a, in which the Al_2_O_3_ film is prepared by atomic layer deposition on a single‐crystalline Si substrate, and the top and bottom electrodes are Pb and p‐doped Si, respectively. Unlike traditional memristors, the state of the proposed devices cannot be regulated by applying voltage. Instead, the read, written, and erase operations of the device are achieved by irradiating UV light or NIR light to the circular Pt top electrode (Figure 7b). With the publication of this work, photonic memristive devices have become one of the research hotpots, while also drawing attention to traditional metal oxides. Until now, a substantial amount of metal oxide materials have proved their potential in the construction of photonic memristive devices, such as ZnO, HfO_2_, In_2_O_3_, Ta_2_O_5_, and WO_3_.^[^ ^2^, ^111^, ^138^, ^139^, ^140^ ^]^ For instance, as a kind of metal oxide, HfO_2_ has been widely applied in traditional electric memristors, while its potential in photonic memristive devices is investigated by Chang et al.^[^ ^141^ ^]^ As shown in Figure 7c, a UV light is selected as the light source to excite the ITO/HfO_2_/TiN‐based memristor, and the *I*–*V* characteristics under UV light exhibit a slight increase compared to the dark condition. The HfO_2_ needs a large forming voltage to initialize the resistive switching behaviors in most cases. Figure 7d depicts the effect of the UV light on the forming voltage of the memristor. The memristor indicates an increase in forming leakage and a decrease in forming voltage under UV light, which can be ascribed to the extra carriers generated by UV light. The inset of Figure 7d shows the calculated average forming voltage for the five devices, which proves that the UV light can effectively reduce the forming voltage by 10%. This work gives a pathway to improve the performance of memristors by applying light.

In addition to hybrid with other materials, the researchers also optimize metal oxides for improving the performance of the memristive devices by doping defects intentionally and controlling the material morphology. As we know, the carriers inside the materials can be excited significantly under the action of light, while doping defects can effectively enhance the ability of the material to capture photo‐generated carriers and extend the lifetime of photo‐generated carriers to strengthen their photo‐response intensity.^[^ ^77^, ^101^, ^142^, ^143^ ^]^ For example, nonstoichiometric CeO_2_ is a kind of photosensitive material in which sensitivity to light mainly depends on the defect energy level located below the conduction band. The photo‐response intensity and photosensitive wavelength can be enhanced and broadened by doping oxygen vacancies. Based on the structure shown in Figure 7e, Li et al. fabricate the polycrystalline CeO_2‐_ *_x_* based‐photonic memristive device.^[^ ^7^ ^]^ Thereinto, CeO_2‐_ *_x_* is the crucial resistive switching functional layer, and the ultrathin AlO*_y_* layer is produced by the oxidation reaction. The Schottky barrier can be formed at the interface between CeO_2‐_ *_x_* and AlO*_y_*. When the UV‐light strikes the devices, the oxygen vacancies within the interfacial CeO_2‐_ *_x_* film adjacent to the AlO*_y_* layer can be further exited by photons, leaving more positively charged oxygen vacancies at the interface and resulting in the reduction of interfacial barriers, which ultimately reduces the conductance of the device (Figure 7f). Additionally, the nonstoichiometric CeO_2‐_ *_x_* has stronger adjustability to light compared with CeO_2_. The excited oxygen vacancies concentration at the interface is significantly increased under strong light intensity, leading to the diverse current responses. Similar to the CeO_2‐_ *_x_* material, the enhanced photo‐response intensity effect by introducing defects is also found in other metal oxide materials, such as SiO_2_,^[^ ^137^ ^]^ WO_3_,^[^ ^139^ ^]^ and MnO*_x_*.^[^ ^140^ ^]^ Another method to improve the photo‐response intensity is controlling the morphology of the material. As we know, the specific surface area of materials is a pivotal factor in the optical absorption coefficient. In brief, the optical absorption coefficient of the same material increases with the specific surface area. Metal oxide is a kind of material that can easily control its morphology, and a large number of reports have reported its simplicity in preparing high specific surface area nanorods or nanosheets. For these reasons, metal oxide nanorods have been applied to photonic memristive devices. Especially, the ZnO nanorods are the first batch materials applied to photonic memristive devices due to the facile and diverse preparation methods. Representatively, Yong et al. prepare the Au/ZnO nanorods/FTO structured photonic memristive devices by hydrothermal method, of which state transition from volatile to nonvolatile can be tuned by applying light.^[^ ^144^ ^]^ In the same way, Guo et al. replace the top electrode Au with Pt to construct the photonic synapse based on the ZnO nanorods (Figure 7g).^[^ ^145^ ^]^ Note that the mechanism for both devices is the same although the electrode is different. Under the dark condition, the ZnO nanorods can absorb the O^2+^ ions in the air due to the large specific surface area, which reduces the free‐moving oxygen vacancies concentration inside the ZnO nanorods. While the desorption of O^2+^ ions on the surface of ZnO nanorods can be promoted by applying light leading to an increase in device conductance (Figure 7h). Based on the above research, Bandopadhyay et al. also investigate the potential of single ZnO nanorods applied in photonic memristive devices, which demonstrates that light can promote the separation of internal electron‐hole pairs to enhance the photogenerated‐current in addition to the weakening effect of adsorbed oxygen on the surface.^[^ ^146^ ^]^ Until now, the photo‐induced resistance switching behavior is also found in MnO*_x_* nanorods,^[^ ^140^ ^]^ WO_3_ nanowires (NWs),^[^ ^147^ ^]^ and others.^[^ ^148^, ^149^ ^]^ We believe that novel approaches of selecting metal oxide to enhance the specific surface area and the light response strength can provide new ideas for constructing emerging photonic memristive and memristive‐like devices.

Briefly, metal oxides are the typical materials for constructing photonic memristive devices. On the one hand, metal oxides are the most widespread materials applied in photonic memristive devices due to their wide variety and various morphology. On the other hand, the preparation method of metal oxide is flexible. Applying the complex physical synthesis methods is beneficial to the stability and repeatability of the device, and it can be compatible with traditional complementary metal oxide semiconductor (CMOS) processes to prepare large‐scale arrays, while the facile chemical synthesis methods are beneficial to low cost, small scale research, and application. And, the further advantages including compatibility with other materials and flexibility (doping, controlling the morphology) make the metal oxides have the potential for further development in photonic memristive devices. Unfortunately, metal oxide materials exhibit several inherent weaknesses as extensive studies on photonic memristive devices, such as the relatively low photo‐response intensity, narrow photosensitive wavelength, low detection rate, and rigidity. Based on these reasons, researchers have developed other material systems to explore the application scenarios of photonic memristive devices.

### 3.2 2D Materials

2D materials are layered crystalline solids, of which electrons can move freely in the nanoscale (1–100 nm) in two dimensional.^[^ ^150^ ^]^ 2D materials have been applied in various applications since graphene is successfully prepared in 2009, such as lubricant,^[^ ^151^, ^152^, ^153^ ^]^ triboelectric nanogenerator,^[^ ^154^, ^155^, ^156^ ^]^ field effect transistor,^[^ ^157^, ^158^ ^]^ and wearable devices.^[^ ^159^, ^160^, ^161^, ^162^ ^]^ In particular, graphene serves as the channel materials to apply in the application of photoelectric detection in the year when it is successfully prepared, which proves that graphene has the capability to be light sensitive.^[^ ^163^, ^164^ ^]^ This work provides a prelude to the applications of 2D materials in photonic devices.

**Figure 8:**
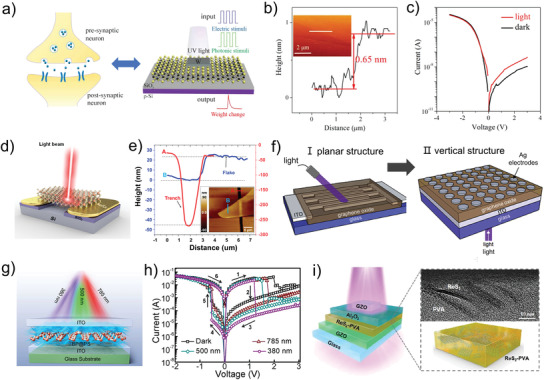
a) Schematic of a biological synapse and photonic synapse based on monolayer MoS_2_. b) AFM profile of monolayer MoS_2_. c) The *I*–*V* curves of the W/MoS_2_/p‐Si device. d) Schematic of free‐standing multilayer MoS_2_ memristive device under light illumination. e) AFM image showing the depth of the trench (200 nm) and the thickness of MoS_2_ (23 nm). f) Schematic of the I) planar structure and II) vertical structure optical resistive switching device consisting of GO deposited between ITO and Ag electrodes. g) Schematic showing light modulation of the BP@PS memristive device. h) *I*–*V* curves of the BP@PS memristive device modulated by different wavelengths. i) Schematic of the photonic memristive device structure with the ReS_2_‐PVA nanocomposite. (a–c) Reproduced with permission.^[^ ^99^ ^]^ Copyright 2018, Wiley‐VCH. (d, e) Reproduced with permission.^[^ ^176^ ^]^ Copyright 2021, American Chemical Society. (f) Reproduced with permission.^[^ ^128^ ^]^ Copyright 2019, Elsevier B.V. (g, h) Reproduced with permission.^[^ ^178^ ^]^ Copyright 2020, American Chemical Society. (i) Reproduced with permission.^[^ ^179^ ^]^ Copyright 2021, American Chemical Society.

The development of 2D materials in the field of memristors has a long history due to the characteristics that are beneficial to the resistance switching behaviors, such as large specific surface area, adjustable band‐gap, and high mobility.^[^ ^165^, ^166^, ^167^ ^]^ A large number of researchers have found the resistive switching behavior in 2D materials, such as graphene and its derivatives,^[^ ^128^, ^168^, ^169^ ^]^ molybdenum disulfide,^[^ ^170^, ^171^ ^]^ tungsten disulfide,^[^ ^172^, ^173^ ^]^ and hexagonal boron nitride,^[^ ^174^, ^175^ ^]^ while the 2D materials are not performed in photonic memristive devices until its concept is proposed in recent years. Even though there are many methods to prepare 2D materials, such as mechanical peel‐off, chemical vapor deposition and sol–gel process, the performances of the 2D materials prepared by various methods are different in memristor due to the limitations of the preparation conditions, which means that the optimization potential of the same 2D material in the photonic memristive and memristive‐like devices is enormous. Guo et al. have been researching memristors based on two‐dimensional materials, including the preparation of ultra‐thin 2D materials and vertical heterojunctions of 2D materials. In recent years, they have successfully developed ultra‐thin direct bandgap MoS_2_ based photonic synapse.^[^ ^99^ ^]^ As shown in **Figure** **8**a, the P‐Si has been selected as the substrate and oxidized on the surface to form an SiO_2_ insulating layer. Then, monolayer MoS_2_ is deposited as a resistive switching layer by chemical vapor deposition (CVD) and transferred to the substrate. Finally, the device is constructed by sputtering the W as the top electrode. As shown in Figure 8b, the thickness of the monolayer MoS_2_ is only 0.65 nm. A high self‐rectification phenomenon is expected owing to the p–n heterostructure between P‐Si substrate and monolayer MoS_2_, resulting in a photodiode‐like behavior. Significantly, the reverse‐biased current under illumination is remarkably enhanced compared with the *I*–*V* curve in darkness, and after turning off the illumination, a persistent photocurrent phenomenon can be observed, as shown in Figure 8c. Regarding these behaviors, the monolayer MoS_2_ plays a pivotal role in generating high light sensitivity. In detail, the ultrathin monolayer structure of the MoS_2_ leads to the enhancement of the optical absorption coefficient, and the high surface‐to‐volume ratio of MoS_2_ results in the increased trap sites from the dangling Si—O bonds at the MoS_2_/SiO_2_ interface, which leads to the increase of the relaxation time of the excitons and the increase of photogenerated‐current. Noting that the conductance variation of the MoS_2_ based photonic synapse is small even though the synaptic plasticity can be realized under UV light. Considering the vulnerability of devices under high‐intensity light, the applied light intensity is only 0.11 mW cm^−2^, which is not enough to produce a large conductance variation. In addition, the complexity and unstability for preparing the large‐scale monolayer MoS_2_ give rise to the limitations of the device in artificial synapse applications. A free‐standing multilayer MoS_2_‐based photonic memristive device is proposed by Adachi et al.^[^ ^176^ ^]^ Instead of optimizing the thickness of the MoS_2_ layer, this group utilizes the simple mechanical exfoliation and transfer methods to fabricate the free‐standing multilayer MoS_2_, and the yield of the preparation process can be over 90% (≈93%), which greatly reduces the complexity of the preparation. Figure 8d shows the schematic diagram of the free‐standing multilayer MoS_2_‐based photonic memristive device. A surface atomic force microscope (AFM) profile of the MoS_2_ is performed and the results are shown in Figure 8e, in which the thickness of the multilayer MoS_2_ is observed to be 23 nm. By applying the light to the free‐standing MoS_2_ channel, the ON/OFF ratio of the memristor can be increased from ≈10^3^ to ≈10^5^. Furthermore, the conductance variation of the memristor can increase from 40 to 190 nA (an increase of ≈150 nA) with a light of 3.08 mW intensity and 10 s duration, which is larger than the devices developed by Guo et al. and enough to identify various resistance states. Generally, several photonic memristive devices composed of MoS_2_ film have been proposed, proving the reliability of MoS_2_ applied in the photonic memristive devices.^[^ ^72^, ^99^ ^]^

The graphene derivatives are utilized earlier and more widely in photonic memristive devices compared with MoS_2_. Graphene cannot be directly served as a resistive switching layer due to the zero‐band gap characteristic. However, the unique chemical activity of graphene makes its derivatives numerous and facile to hybrid with other materials. As a kind of graphene derivative, graphene oxide is the first kind of 2D material applied in an photonic memristive devices. Based on graphene oxide GO materials, Kemp et al. construct the planar and vertical Ag/GO/ITO photonic memristive devices by drop‐casting for the first time (Figure 8f).^[^ ^128^ ^]^ The function of photoelectric operation to memristor can be realized in both I) planar and II) vertical structures, proving the flexibility of graphene oxide in practical applications. The photogenerated‐current response of the planar devices to UV light is in the order of microamps, while the response to red, green and blue (RGB) light is only nanoamps, which indicates the sensitivity of GO to the UV‐wavelength region. In particular, the response of planar devices to UV light is irreversible due to the partial reduction of GO, while the reduction cannot occur under RGB light. Furthermore, this group prepares the vertical devices to improve the light‐induced ON/OFF ratio. Although the current response of light to vertical devices is only one‐tenth of that of planar devices, the light‐induced ON/OFF ratio of the vertical devices is larger than planar devices under UV light as a result of the low initial conductance of vertical devices. Besides constructing various device structures, the versatility of GO in photonic memristive devices can also be reflected in their flexibility and scalability. For example, combining with the TiO_2_ nanoparticles, Liu et al. construct a graphene oxide‐based flexible photonic memristive device.^[^ ^165^ ^]^ The TiO_2_ nanoparticles are physically adsorbed on the surface of GO as a photocatalyst, which increases the light response capability without affecting the flexibility of the devices.

For the sake of improving the performance of the photonic memristive device, several emerging 2D materials and hybrid film formation approaches are developed.^[^ ^177^, ^178^, ^179^ ^]^ Possessing the broad light absorption and natural direct bandgap, black phosphorus (BP), which is a class of emerging 2D materials, is beneficial for constructing photonic memristive devices, but the fragmented sheet‐like structure makes it difficult to construct active layers alone. The BP‐based hybrid systems, such as ZnO/BP heterojunction and BP‐polymer nanocomposite, are the effective way to solve this issue. BP nanosheets coated with polystyrene (BP@PS NSs) are synthesized and proposed to select as the active layer in the ITO/BP@PS/ITO photonic memristive device.^[^ ^178^ ^]^ During the preparation, PS is dissolved in chloroform and mixed with the BP NSs ultrasonically to complete the surface modification of BP, then the PS‐coated BP NSs are spin‐coated on the ITO substrate to form an active layer. Figure 8g shows the schematic diagram of the ITO/BP@PS/ITO photonic memristive device, in which the BP is evenly wrapped by PS and forms a continuous and flat film. Three kinds of light (380 nm, 500 nm, and 785 nm) are employed separately to acquire the *I*−*V* curves under simultaneous electrical and optical stimulations, and the measurement results are shown in Figure 8h. It can be seen that the ON/OFF ratios of the photonic memristive device exhibit a slight enhancement trend with the increase of the wavelengths, and the setting and resetting voltages also depend on the wavelengths. BP is a promising resistive switching material in photonic memristive devices, and the novel device configuration provides insights into the development of multifunctional microelectronic devices based on 2D materials. Furthermore, a metal oxide layer is added to the hybrid film systems to exert the photoresponse properties of the 2D material. Recently, a photonic memristive device structured by Ga‐doped ZnO (GZO/Al_2_O_3_/ReS_2_‐ployvinylalcohol (PVA)/GZO (shown in Figure 8i) is proposed by Yun et al., in which the GZO serves as the electrode, while the metal oxide layer Al_2_O_3_ acts as the protective layer and the ReS_2_‐PVA is the active layer.^[^ ^179^ ^]^ The 2D material ReS_2_ has a sheet‐like structure similar to BP, thus PVA is used as the exfoliation agent during the preparation process to form the matrix of the ReS_2_‐PVA nanocomposite film. When the light is radiated onto the device, a large number of electrons are generated in the ReS_2_ nanosheets, and they are captured in the defect‐level trap sites, finally resulting in the photo‐response. Thereinto, the protective layer Al_2_O_3_ acted as a barrier to eliminate unexpected early electrical breakdown and high off‐current levels, significantly improving the uniform of the resistive switching behaviors. This work proposes a cost‐effective, environmentally friendly, and applicable to various TMD nanomaterials method to prepare the 2D materials‐based photonic memristive device.

The types of 2D materials applied in photonic memristive and memristive‐like devices are scarce, which can be attributed to the instability and difficulty in preparation. However, given the outstanding performance of 2D materials in traditional electronic memory devices and the unique properties in light tunability, we believe it has great potential for applications in photonic memristive and memristive‐like devices in the future. For example, the high photo‐response intensity ensures the low power consumption and differentiation of various resistance states, and the optical tunable bandgap makes the devices the ability to adjust the ratio of the devices. In summary, exploiting more kinds of two‐dimensional materials that can be applied in photonic memristive and memristive‐like device will always be a research hotspot.

### 3.3 Perovskite

ABX_3_‐type perovskites, which are regarded as versatile functional materials, have been receiving increasing attention in various applications due to the light absorption coefficient, slow rates of nonradiative charge recombination, and unique lattice structure.^[^ ^180^, ^181^, ^182^, ^183^, ^184^ ^]^ As a matter of fact, the researchers have found obvious resistive switching behaviors in various perovskite materials, such as SrTiO_3_,^[^ ^185^ ^]^ BaTiO_3_,^[^ ^186^ ^]^ CH_3_NH_3_PbI_3_,^[^ ^187^ ^]^ CH_3_NH_3_PbBr_3_,^[^ ^188^ ^]^ and CsPbI_3_.^[^ ^189^ ^]^ Based on the excellent performance in electric‐induced resistive switching and photosensitivity, the perovskite materials have been applied in photonic memristive and memristive‐like devices recently.

**Figure 9:**
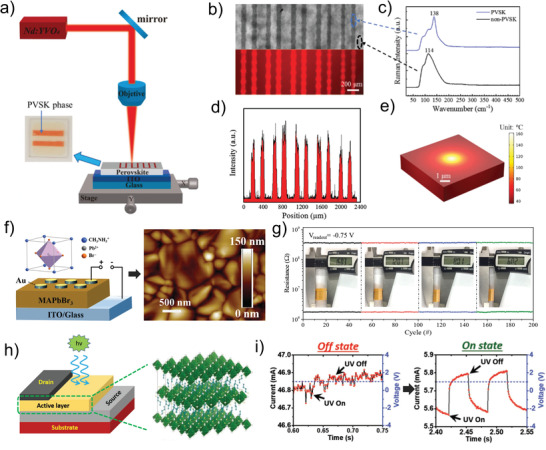
a) Schematic illustration Laser direct writing (LDW) of the perovskite film assisted by photothermal effect. The inset shows the photo of a perovskite thin film with two stripes converted to the PVSK phase through LDW. b) Optical (top) and fluorescent (bottom) microscopy images of the micro‐lines pattern by LDW. c) Measured Raman spectra at different positions. The two distinct spectra correspond to the non‐PVSK phase (black curve) and the PVSK phase (blue curve). d) Line‐scan PL intensity of the fluorescent image in (b). e) COMSOL Multiphysics thermal simulation of heat distribution induced by a laser beam (9 mW) with a spot size of 1 µm. f) Schematic diagram of the switching device with Au/MAPbBr_3_/ITO structure, and AFM image of the perovskite layer surface. g) Endurance performance of the flexible RS device measured with various bending radius: 8, 6, 4, and 3 mm (from left to right). h) Proposed two‐terminal simple photonic memristive device with drain (NiO), active layer [(C_4_H_9_NH_3_)_2_PbBr_4_], and source (ZnO) and the schematic presentation of the three vertically stacked layers of the (C_4_H_9_NH_3_)_2_PbBr_4_. i) Experimentally measured absence of photo‐response under this condition‐designated OFF state and ON state. (a–e) Reproduced with permission.^[^ ^197^ ^]^ Copyright 2018, Wiley‐VCH. (f, g) Reproduced with permission.^[^ ^100^ ^]^ Copyright 2018, Wiley‐VCH. (h, i) Reproduced with permission.^[^ ^205^ ^]^ Copyright 2018, Wiley‐VCH.

As the most typical one in the type of perovskite materials, inorganic oxide perovskites have promising potential for photonic memristive and memristive‐like devices as a result of the reliable film and fast speed of ion diffusion.^[^ ^190^, ^191^ ^]^ However, the inorganic oxide perovskites need doping to improve their response toward the light in virtue of the weak light absorption coefficient. Li et al. present an artificial photonic synapse based on the Nb:SrTiO_3_. The doping of Nb increases the density of defects in the SrTiO_3_, which enhances the trapping ability of the film to photo‐generated carriers, thereby realizing the plasticity of the photonic synapse.^[^ ^65^ ^]^ Besides the complexity of the fabrication process caused by the doping and the inertness property toward the light, the precise and expensive preparation processes (physical preparation, high‐temperature requirements) also lead to the limitations of its application in the photonic memristive devices. Recently, inorganic halide perovskites have become the focus materials for the construction of photonic memristive and memristive‐like devices by means of the fast speed of ion diffusion, various prepared processes, and high light absorption coefficient.^[^ ^192^, ^193^, ^194^, ^195^, ^196^ ^]^ The inorganic halide perovskites, which can be deemed as the derivatives of perovskites by replacing the X site element in the ABX_3_ with halide elements, possess unique advantages in generating resistive switching behavior because the photo‐generated electron‐holes pairs are susceptible to chemical reactions with halide elements. An inorganic halide perovskites‐based photonic memristive device is proposed by Chen et al., where Cs_4_PbBr_6_ serves as the active layer and PEDOT:PSS acts as the adhesion layer.^[^ ^105^ ^]^ In terms of preparation methods, the Cs_4_PbBr_6_ layer is prepared by two facile and low‐cost processes, including the spin coating and chemical solution methods. For the fabrication of the Cs_4_PbBr_6_ layer, the PbBr_2_ precursor solution is spin‐coated on the PEDOT:PSS film, then immersed in a solution with CH_3_OH. Finally, the preparation of the Cs_4_PbBr_6_ layer is completed after annealing. The compound reaction of photo‐generated electrons and Br vacancies is more intense under illumination, which leads to significant photo‐induced resistive switching behaviors. Based on similar inorganic halide perovskite CsPbIBr_2_ film, a novel optical switch (memristive‐like device) is proposed by Lin et al. For preparing the device, the CsI, PbBr_2_, is separately placed into two crucibles and simultaneous sublimed, and vapor‐deposited onto the pre‐patterned substrate.^[^ ^197^ ^]^ The mixed halide inorganic CsPbIBr_2_ film can be obtained by tuning the evaporation rates of the two sources. Distinct from photo‐generated carriers produced by using perovskites, the CsPbIBr_2_ can undergo a phase change process between optically absorbing perovskite (PVSK) phase to non‐perovskite (non‐PVSK) phase under the action of the laser. As shown in **Figure** **9**a, a near‐NIR (1064 nm, Nd:YVO_4_) laser for laser direct writing (LDW) is applied to the non‐PVSK phase CsPbIBr_2_ film, and it can be observed that the optical and fluorescent microscopy images of the CsPbIBr_2_ after laser writing have obvious changes (Figure 9b). The phase transition is also characterized by the photoluminescence spectroscopy (PL), Raman spectra and proved by a multiphysics thermal simulation (Figure 9c–e). Benefitting from the stability and controllability of the phase transition, this device exhibits great write‐read‐erase cycle endurance and data retention capability without obvious performance degradation. Recently, the organic‐inorganic hybrid halide perovskite‐based photonic memristive and memristive‐like devices are developed by replacing the A‐site element in the perovskite molecular formula with organic CH_3_NH_3_(MA) macromolecules.^[^ ^198^, ^199^ ^]^ The organic–inorganic hybrid halide perovskites not only have the chemical stability of inorganic materials but also have excellent physical and structural properties of organic materials. Considering the effect of light absorption coefficient and lattice stability of the organic–inorganic hybrid halide perovskites, Wu and co‐researchers construct the photonic memristive devices based on organic–inorganic hybrid halide perovskite MAPbBr_3._^[^ ^100^ ^]^ The structure and surface AFM characterizations of the MAPbBr_3_‐based device are shown in Figure 9f. To reduce the surface roughness of the MAPbBr_3_ layer and weaken the hot spot effect that can affect the stability of the devices, a novel two‐step sequential vapor conversion technique is implemented, which consists of preparing the PbBr_2_ layer by thermal evaporation and placing the substrate on a hot plate with MABr powder to form the MAPbBr_3_ layer. When the light strikes the MAPbBr_3_‐based devices, the drift of the photo‐generated carriers in the perovskite layer under the effect of the built‐in electric field leads to the increase of the photocurrent, reducing the resistance of HRS and LRS. Moreover, the researchers demonstrate the potential of the MAPbBr_3_‐based devices for flexible devices due to the flexibility of MAPbBr_3_. As shown in Figure 9g, the performance of MAPbBr_3_‐based devices remains stable after 200 bends, indicating the application potential of organic–inorganic hybrid halide perovskites in flexible photonic memristive devices.

2D halide perovskites with high molecular stability and photo‐response intensity, which have a general formula of (L)_2_(SMX_3_)*_n_*_+1_MX_4_, where L, S, M, and X represent long‐chain organic cations, short‐chain organic cations, divalent metal cation, and halide, respectively, have been prepared by reducing the dimension of the perovskite molecular structure to exert the light response‐ability of halide perovskites.^[^ ^200^, ^201^, ^202^, ^203^, ^204^, ^205^ ^]^ Aiming to the problem that the introduced cations cannot match the typical perovskite cubic octahedral structure, the dimensionality reduction of the perovskites is implemented to adapt to the bonding of long organic molecular chains. Typically, the (C_4_H_9_NH_3_)_2_PbBr_4_, as a kind of typical 2D halide perovskite material, has been proposed and applied in visual photonic memristive devices by Kim et al.^[^ ^205^ ^]^ As shown in Figure 9h, the structure of the (C_4_H_9_NH_3_)_2_PbBr_4_‐based photonic memristive devices from top to bottom are the ZnO source electrode prepared by magnetron sputtering, the (C_4_H_9_NH_3_)_2_PbBr_4_ active layer prepared by sol–gel method, and the NiO drain electrode prepared by magnetron sputtering, respectively. For the sake of reducing the surface roughness and hot spot effect, a two‐step sequential growth method is performed to prepare the (C_4_H_9_NH_3_)_2_PbBr_4_ active layer. For the sake of reducing the surface roughness and hot spot effect, a two‐step sequential growth method is performed to prepare the (C_4_H_9_NH_3_)_2_PbBr_4_ active layer. Unlike the two‐step sequential vapor conversion technique proposed by Wu and co‐researchers, this method utilizes two‐step spin coating processes and a one‐step annealing process, which efficiently improve the quality of the film. Furthermore, the (C_4_H_9_NH_3_)_2_PbBr_4_‐based photonic memristive devices exhibit a series of unique phenomena under the combination of voltage and light stimulation, as shown in Figure 9i. In detail, when the device is exposed to UV light, the recombination of photo‐generated carriers and dark current can be suppressed by applying a negative voltage, which results in the reduction of conductance value (corresponding to the ON state). While the recombination of photo‐generated carriers and dark current can be promoted by applying the negative voltage, leading to device inertness toward the light (corresponding to the OFF state). Worth noting that the presence of Pb elements in (C_4_H_9_NH_3_)_2_PbBr_4_ gives rise to environmental pollution, even though the (C_4_H_9_NH_3_)_2_PbBr_4_ exhibits various excellent photoelectronic performances.

As mentioned above, the merits of perovskite materials in terms of preparation, light absorption coefficient, and photosensitive wavelength exhibit promising potential in photonic memristive and memristive‐like devices. However, there are still many obstacles that remain unresolved even though the application of perovskite in photonic memristive and memristive‐like devices has been optimized for a long time. For example, the problems of the hot spot effect induced by rough perovskite surface, the variability of the lattice structure, and instability under electric field require researchers to seek optimal preparation methods and develop stable perovskite materials.

### 3.4 Quantum Dots

**Figure 10:**
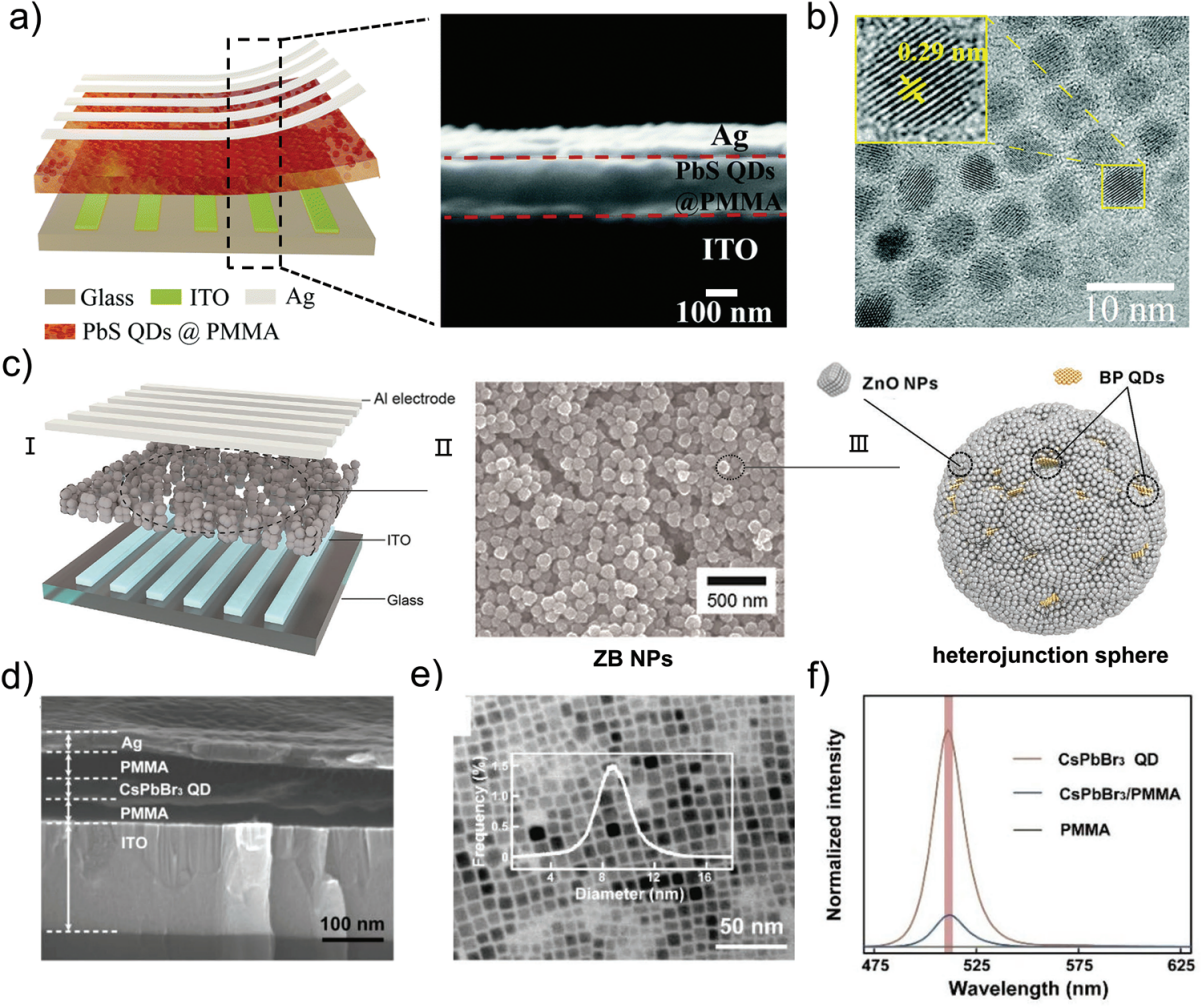
a) Device structure diagram and the cross‐sectional SEM image of the Ag/PbS QDs@PMMA/ITO memory device. b) TEM image of PbS QDs. c) I) Schematic of ZB hybrid NPs‐based photonic RRAM. II) Top‐viewed SEM image of ZB NPs film and III) the 3D image of ZB hybrid NPs. d) Schematic illustration of the CsPbBr_3_ QD‐based photonic RRAM device. e) TEM image of the CsPbBr_3_ QDs, inset is the diameter distribution of the QD. f) Steady‐state one‐photon‐excited (ope) (*λ*_ex_ = 400 nm) PL spectra. (a, b) Reproduced with permission.^[^ ^106^ ^]^ Copyright 2020, Royal Society of Chemistry. (c) Reproduced with permission.^[^ ^203^ ^]^ Copyright 2018, Wiley‐VCH. (d–f) Reproduced with permission.^[^ ^90^ ^]^ Copyright 2018, Wiley‐VCH.

Quantum dot is a kind of 0D material with a size of <30 nm and its applications in semiconductor devices are gradually increasing.^[^ ^206^, ^207^, ^208^, ^209^ ^]^ In recent years, quantum dots have demonstrated preeminent performance in various applications, such as photodetectors,^[^ ^136^, ^210^, ^211^ ^]^ light‐emitting diodes (LEDs),^[^ ^212^, ^213^, ^214^ ^]^ and fluorescent dye,^[^ ^215^, ^216^ ^]^ which can be ascribed to the facile chemical preparation methods, precise and controllable grain size, and quantum effect. Meanwhile, quantum dots are also extensively applied in traditional memristors based on above mentioned superior merits.^[^ ^217^, ^218^ ^]^ Most researchers have focused on the tunable bandgap and precisely controllable concentration/thickness characteristics of quantum dots to regulate the performance of memristors, ignoring the natural UV fluorescence properties. Recently, the high photoluminescence quantum yield, high photochemical stability, and tunable spectral characteristics in virtue of the unique structure, fluorescence, and other characteristics have made quantum dots “star material” for the preparation of photonic memristive devices. At present, a large number of quantum dots have been proved to have the potential to be applied in photonic memristive devices, such as BP QDs,^[^ ^219^ ^]^ PbS QDs,^[^ ^106^ ^]^ CdSe/ZnS QDs,^[^ ^220^ ^]^ N‐GO QDs,^[^ ^221^ ^]^ and  caron QDs.^[^ ^222^, ^223^ ^]^ Due to the limitations of individual film formation, quantum dot materials need to be mixed with other materials to construct devices, but it increases flexibility in quantum dot applications. The hybrid of polymers with quantum dots is the most typical method to overcome these obstacles. As shown in **Figure** **10**a, Zhang et al. fabricate a photonic memristive device based on the hybrid layer of PbS QDs and PMMA, where the smooth hybrid layer breaks through the roughness limitation of quantum dot film‐forming individually (Figure 10b).^[^ ^106^ ^]^ PMMA, as a kind of outstanding surface organic ligand material, can be facile hybridized with quantum dots to prepare the smooth surface, while the PbS QDs serve as the superior active layer on account of the simple and diverse chemical synthesis methods and stability after hybridizing with polymers. Extraordinary, the PMMA layer in the proposed device cannot produce a light response but act as a barrier layer to reduce the leakage current. Because of the wide absorption wavelength and narrow emission wavelength of PbS QDs under UV light, the strong photo‐response of the proposed device under UV light can be achieved, however, the narrow photo‐response limits its applications.

Moreover, quantum dots exhibit excellent performance in photonic memristive devices after combining with metal oxides. This kind of device can not only exert the superiority of quantum dots in high response to UV light but also widen the photo‐response band and realize the dual‐band response by combining with specific metal oxides. The simplest hybrid method is the construction of heterojunctions, which can properly solve the poor performance caused by non‐uniform film and chemical activity at the edges. Based on this way, Ruan et al. construct a photonic memristive device based on a mixed functional layer of ZnO nanoparticles and BP QDs.^[^ ^203^ ^]^ The device structure and BP QDs/ZnO nanoseed heterostructure are shown in Figure 10c. When the device is exposed to UV light, a large number of photo‐generated carriers are produced in the ZnO/BP heterojunction in virtue of introducing the ZnO, enhancing the resistive switching behavior. As we know, the BP QDs is a kind of material that sensitive to UV‐light, while the photo‐response wavelength can be expanded from UV‐light (365 nm) to near‐NIR (785 nm) by introducing the ZnO nanoparticles. The wide‐band response enables the device to adapt to the illumination of various bands and expands its application field. The photonic memristive devices with dual‐band response capability are also applied to regulate the gradient conductance (artificial synapse). Different from the wide‐band response, the dual‐band response is sensitive to two specific wavelengths of light. Typically, the ZnO/PbS QDs‐based photonic memristive devices proposed by Zhou et al. realize the dual‐band response of UV and NIR light, which can simulate different synaptic functions to form a novel artificial neural network.^[^ ^64^ ^]^ When UV and NIR lights irradiate the device, the current of the device shows the trend of enhancement and weakening corresponding to the stimulation and suppression of synapses. It can be seen that most of the above oxides combined with quantum dots are based on ZnO because of the ease of ZnO preparation and high light response efficiency. We believe that similar materials, such as SnO_2_, TiO_2_, also have the potential in constructing photonic memristive devices.

To improve performance, a perovskite quantum dots‐based photonic memristive device is proposed to exert the structure merits of perovskite with the size superiority of quantum dots. Limited by the preparation methods, the perovskite quantum dots are basically inorganic halide perovskite structures. Being mixed with PMMA, the Ag/PMMA/CsPbBr_3_ QDs/PMMA/ITO structured photonic RRAM are constructed by Han et al.^[^ ^90^ ^]^ The cross‐sectional SEM of CsPbBr_3_ QDs‐based photonic RRAM is exhibited in Figure 10d. The average particle size of perovskite quantum dots prepared by a simple chemical solution method is about 8 nm, as the transmission electron microscope (TEM) image is shown in Figure 10e. As we mentioned in the perovskite section, inorganic halide perovskites exhibit poor flexibility as a result of the rigid structure, and the perovskite quantum dots have the potential for flexible devices owing to the simplification hybrid with polymers. The edge coordination of organic toward perovskite quantum dots can weaken the response‐ability to light to a certain extent, which limits its practical application in photonic memristive devices (Figure 10f).

In brief, quantum dots have been applied as a kind of material for photonic memristive devices with great potential by researchers. So far, the quantization effect caused by the structure makes the quantum dots have the strongest light response at a specific UV wavelength, which is conducive to the realization of a large photo‐induced switching ratio of the device. The advantages of facile preparation methods, ease to mix with other materials, and the expandable photo‐response band makes quantum dots have good development prospects in light‐responsive devices. However, with the deepening of research, the defects of quantum dots are also revealed. UV light is extremely insensitive to other wavelengths of light, and when the quantum dots are used alone as the dielectric layer, the film performance is extremely uneven and affects device performance. These reasons limit the application of quantum dots in photonic memristive devices in some ways.

### 3.5 Upconverting Nanoparticles

**Figure 11:**
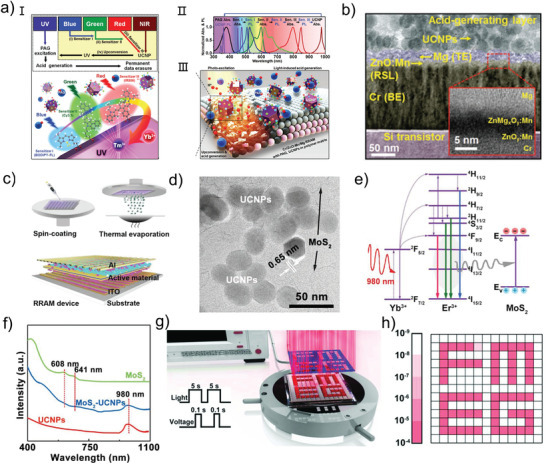
a) Design of multi‐dye‐sensitized UCNPs and integration with ultrathin nonvolatile memory for advanced information security. I) Design of multi‐dye‐sensitized UCNPs for wide‐range photo‐absorption and upconversion. II) Normalized absorption and photoluminescence spectra of the UCNP, sensitizers (I), (II), (III), and normalized absorption spectrum of the PAG. III) schematic illustration of the photo‐induced unrecoverable data erasure in the ultrathin nonvolatile memory device with UCNPs. b) Cross‐sectional TEM image of the integrated system (RRAM on the Si transistor coated with UCNPs/PAG/PEO layer). c) Schematic illustration and cross‐section scanning electron microscopy image of the photonic memristive device. d) TEM image of as‐prepared MoS_2_‐UCNPs nanocomposite. e) Simplified energy level diagram describing upconverting PL process and MoS_2_ excitation process. f) Absorption spectrum of the UCNPs, MoS_2_, and MoS_2_‐UCNPs nanocomposite. g) System integration of the 13 × 13 array with light signal. h) The light signals are stored in the 13 × 13 array. (a, b) Reproduced with permission.^[^ ^230^ ^]^ Copyright 2016, Wiley‐VCH. (c–h) Reproduced with permission.^[^ ^49^ ^]^ Copyright 2018, Wiley‐VCH.

As a novel kind of photoelectric material, upconverting nanoparticles (UCNPS) have exhibited promising application and exploration potential in photonic devices in light of the high optical absorption coefficient, fast photon emission rate, robust photochemical stability, large anti‐stokes shift, and sensitivity to near‐NIR light.^[^ ^224^, ^225^, ^226^, ^227^, ^228^, ^229^ ^]^ In fact, applications of UCNPS in photonic memristive devices are relatively rare, which can be ascribed to the roughness in film formation and the complexity of energy conversion. Most of the practical applications in photonic memristive devices are based on multi‐dye‐sensitized UCNPS owing to the requirements of energy transfer between multiple ligands to form an energy gradient. A photonic memristive system composed of a UCNPs/photo‐acid‐generator (PAG)/poly(ethylene oxide) (PEO) photodetector and a Cr/ZnO: Mn/Mg memristor is proposed by Hyeon et al., which is the first report to illustrate the potential of the UCNPs application in photonic memristive device.^[^ ^230^ ^]^ As shown in **Figure** **11**a‐I, the presence of Yb and Tm in *β*‐NaYF^4+^:20%Yb, 0.5% Tm UCNPs enables the photon energy to be up‐converted to the NIR emission band. Figure 11a‐II exhibits the photoluminescence spectra of the multi‐sensitized UCNPs, which indicates that three ligands and the UCNP itself cover the entire visible and NIR spectral range for the photo‐absorption process. Especially, the proposed device reveals a chemical destruction phenomenon under emergencies. As shown in Figure 11a‐III, the ultrathin ZnO:Mn resistive switching layer rapidly disappears in an integrated system due to the acidic environment produced by the light illumination. Unfortunately, the UCNPs/PAG/PEO structure only acts a photosensitive function in series with Cr/ZnO:Mn/Mg memristor instead of resistive switching active layer, as depicted in the cross‐sectional TEM image of the photonic memristive system (Figure 11b). Based on the previous research, Zhou et al. realize the resistive switching behaviors in MoS_2_‐NaYF_4_:Yb^3+^, Er^3+^ UCNPs‐based photonic memristive device.^[^ ^49^ ^]^ The structure of the proposed device and TEM characteristic results of UCNPs and MoS_2_ nanosheets are shown in Figures 11c,d, respectively. The Yb^3+^ plays a vital role in the response to the NIR, which can be proved by the schematic diagram of the energy up‐conversion and absorption spectrum of the MoS_2_‐NaYF_4_:Yb, Er^3+^ UCNPs (Figure 11e). After combining with MoS_2_, the NIR photon energy absorbed by Yb^3+^ is converted into a high energy level, then the photonic energy releases NIR wavelength energy near the Er^3+^. Followed the released energy is partially absorbed by MoS_2_, and, the photon emission wavelength of the MoS_2_‐NaYF_4_:Yb^3+^, Er^3+^ UCNPs is widened. As the absorption spectrum of the nanocomposite is shown in Figure 11f, the multi‐dye‐sensitized UCNPS has significant advantages in the regulation of memristive performance in the range of the NIR band. Furthermore, a 13 × 13 array is designed to perform the bioinspired vision system. As shown in Figure 11g, a 980 nm light with the intensity of 500 mW cm^−2^ is applied to the array through a designed mask, and the resistance states of each cell are read by a reading voltage of 0.5 V. As a result, the resistive value of the illuminated cell is about 10^4^ Ω, which is significantly larger than the resistive value of other cells (10^6^ Ω). Under the assistance of designed illumination, the array displaying the stored information (the characters “FMEG”) is written by the light, as shown in Figure 11h.

The transition from low to high energy can be achieved in UCNPs by utilizing their unique photosensitive properties, which exhibit great potential in photonic memristive devices, especially in NIR band response memristive devices. Additionally, multi‐dye‐sensitization of UCNPs can dramatically broaden their photon absorption region from only the NIR range (975 nm) to the entire visible and NIR range (450–975 nm). In a word, benefiting from the adjustable photon absorption window induced by the sensitization, the UCNPs have promising potential in full‐photo modulated photonic memristive devices.

### 3.6 Others Materials

In addition to the materials mentioned above, there are lots of unique materials that can be applied in photonic memristive and memristive‐like devices, such as biocompatible materials,^[^ ^231^, ^232^ ^]^ ferroelectric materials,^[^ ^233^, ^234^ ^]^ chalcogenide compounds,^[^ ^235^, ^236^, ^237^ ^]^ and optically active polymer.^[^ ^129^, ^130^, ^131^, ^238^ ^]^ However, in view of their relatively infrequent applications and research, we will summarize the characteristics of these materials and briefly introduce them in this chapter.

**Figure 12:**
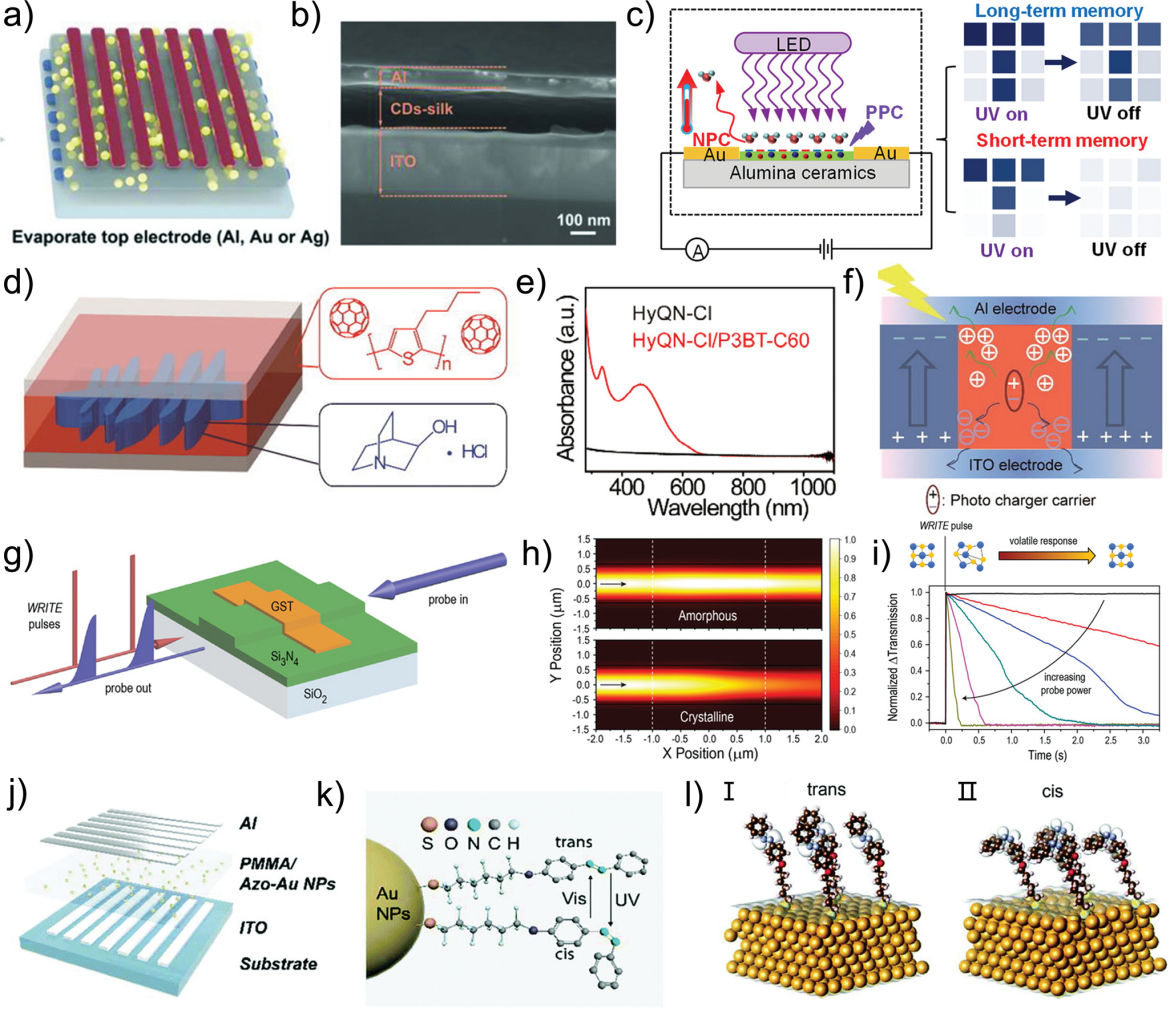
a) Schematic illustration of the CDs‐silk memory device. b) Cross‐sectional SEM image of the device structure. c) Schematic diagram of the device structure and the image mapping of the LTM and STM processes. d) Device scheme of the HyQN‐Cl/P3BT C_60_ blends and chemical structure of ferroelectric (R)‐(−)‐3‐hydroxlyquinuclidinium chloride, C_60_, and poly(3‐butylthiophene) (P3BT). e) Absorption of HyQN‐Cl/P3BT C_60_ blends. f) Device scheme and the photovoltaic switching mechanism in HyQN‐Cl/P3BT C_60_ devices. g) Illustration of the GST‐based PCM and measurement scheme. h) FDTD simulations of the power flow from left to right through the region of GST when GST is in both the amorphous and crystalline states. i) Experimental optical transmission of the device with increasing optical probe power. j) Device structure of Azo‐Au NPs based memristor. k) Schematic of AZO functional Au NPs. l) Schematic diagram of the Azo‐Au NPs I) before and II) after UV light irradiation. (a, b) Reproduced with permission.^[^ ^124^ ^]^ Copyright 2017, Royal Society of Chemistry. (c) Reproduced with permission.^[^ ^232^ ^]^ Copyright 2020, American Chemical Society. (d–f) Reproduced with permission.^[^ ^251^ ^]^ Copyright 2019, Elsevier B.V. (g–i) Reproduced with permission.^[^ ^237^ ^]^ Copyright 2019, Wiley‐VCH. (j–l) Reproduced with permission.^[^ ^238^ ^]^ Copyright 2020, Royal Society of Chemistry.

Recently, materials that are compatible with organisms have attracted the attention of researchers due to their unique bionic properties and harmlessness to the human body. The superior environmental protection of the photosensitive biomaterials showing the degradability and recoverability can alleviate the problem of electronic pollution at the present stage. Until now, various types of biomaterials can be served as the active layer for the fabrication of photonic memristive devices, such as protein,^[^ ^148^ ^]^ polysaccharide,^[^ ^239^ ^]^ nucleic acid,^[^ ^240^ ^]^ and virus.^[^ ^241^ ^]^ Typically, as a kind of biocompatible material, carbon dots (CDs) have emerged as promising materials for photonic applications because of their small size, attractive optical properties, and low preparation cost. The CDs‐silk protein‐based photonic memristive devices is proposed by Roy et al., which is constructed using Al/CDs‐silk/Ag/ITO structure.^[^ ^124^ ^]^ The device structure and the cross‐sectional SEM image are shown in **Figures** **12**a,b, respectively. Under the action of the light, the CDs‐silk protein‐based photonic memristive devices can be regulated between multilevel states. In particular, the excellent biodegradability and biocompatibility of the CDs‐silk can be effectively degraded in the human body, which exhibits potential applications in future implantable electronic devices. Liu et al. exhibit a biocompatible photonic RRAM structured nanofibers self‐assembled with cyclo‐tyrosine‐tyrosine (cyclo‐YY).^[^ ^232^ ^]^ As shown in Figure 12c, the photonic RRAM has a planar structure with Au electrodes. When the LED light is applied to the active layer, the conductance increases because of the photo‐oxidation effects of the cyclo‐YY, indicating the vision sensor potential. By tunning the light parameter, the transition between short‐term visual memory to long‐term visual memory is realized in the image array, as shown in Figure 12c. The biocompatibility of the cyclo‐YY paves a new way to construct the artificial cogni‐retina. In fact, biocompatible materials can achieve the interface between the electronic devices and the biological environment, while it can meet the new requirements of the development and broaden the corresponding biotechnology and semiconductor applications, such as implant chips,^[^ ^242^, ^243^ ^]^ artificial neurons^[^ ^244^, ^245^ ^]^ and electronic skin.^[^ ^246^, ^247^, ^248^ ^]^

Ferroelectric materials have promising potential in traditional non‐volatile memory as a result of the characteristics of ferroelectric phase transition under an electric field.^[^ ^9^, ^249^, ^250^ ^]^ However, traditional inorganic ferroelectric materials are insensitive to light, which limits their application in photonic memristive devices. Recently, Ren et al. combine the organic molecular ferroelectric materials ((R)‐(−)‐3‐hydroxlyquinuclidinium chloride, HyQN‐Cl) with donor‐acceptor polymers (poly(3‐butylthiophene)‐C60, P3BT‐C_60_) to construct the photonic memristive devices, as shown in Figure 12d.^[^ ^251^ ^]^ The ferroelectric material HyQN‐Cl exhibits a semiconductor phase in the initial state. As the absorption spectrum shown in Figure 12e, the HyQN‐Cl/P3BT‐C_60_ films show a broad photoabsorption covering from UV to near‐NIR region, proving the light sensitivity of the proposed RRAM. When light irradiates the device, the HyQN‐Cl can generate photogenerated excitons. Then the P3BT as a donor can capture photo‐generated holes while the acceptor C_60_ can capture photo‐generated electrons, resulting in the emerging of the stray field in the HyQN‐Cl/P3BT C_60_ heterojunction and the transition of the HyQN material to ferroelectric phase (Figure 12f). The proposal of photo‐induced ferroelectric phase transition provides a new idea for constructing photonic memristive devices. In reality, this work is the first photonic memristive device constructed by combining organic molecular ferroelectric materials with donor‐acceptor polymers.

As a kind of fast ion conductor, the chalcogenide compounds have been widely applied in the study of memristors because they can provide a fast migration channel for metal ions. In recent years, some researchers have verified the redox or phase transition process of chalcogenide compounds under light, which has promoted its application in photonic memristive and memristive‐like devices. A typical chalcogenide compound Ag_2_S is a solid‐electrolyte with Ag^+^ and S^2−^ ions, and its conductivity can be significantly excited by a green laser. A photonic memristive device structured by Ag/Ag_2_S/Au is proposed by Massood et al., in which the Ag_2_S film is prepared by a sputtering and sulfurization process.^[^ ^235^ ^]^ The redox potential of the Ag_2_S in presence of illumination induces the reduction in activation energy of the Ag atoms, significantly promoting the formation of conductive filaments. In the very recent work by Bhaskaran et al., a photonic memristive device based on Chalcogenide GeSe_3_ is proposed.^[^ ^236^ ^]^ The device structure is based on metal/GeSe_3_/metal, and the electrode of the device is flexible and configurable, in which the selection of active metal, such as Ag, can promote the formation of the conductive filament. Especially, the movement of the Ag ions in the GeSe_3_ can be coordinated modulated by the optical and electrical signals. At first, the directional migration of Ag ions driven by an appropriate voltage can form the Ag conductive filaments, while under the optical case, the Ag electrode spontaneously dissolves into Ag clusters which are then photomigrated and photodeposited onto the GeSe_3_ film. Then, the filaments are formed under mixed effects, such as photovoltaic effect, photothermal effect, and redox. These results give a broad explanation between different physical phenomena in the GeSe_3_, and successfully emulate three‐factor neo‐Hebbian plasticity owing to its optoelectronic synergistic regulation. In addition to exploiting the superior optical properties of chalcogenides, the phase changes of chalcogenides under light are a stable and reliable pathway to implement the photonic memristive device. A continuing focus of chalcogenides research is on the well‐known chalcogenide alloy Ge_2_Sb_2_Te_5_ (GST) due to its excellent electrical and phase change properties. A tunable volatility optical PCM (memristive‐like device) based on GST is constructed by Bhaskaran et al., of which structure is exhibited in Figure 12g.^[^ ^237^ ^]^ The device is prepared on a passivated layer of indium‐tin‐oxide (ITO) on top of an Si_3_N_4_ photonic waveguide, and a thick strip of GST is finally deposited by sputtering to form the device. Optical WRITE pulses are applied to trigger phase transitions, at the same time, an optical probe is used to continuously monitor the transmission state of the GST. The FDTD simulations were performed to calculate the optical transmission through the waveguide. As shown in Figure 12h, the top‐down view of the power flow proves that there is a significant reduction in the waveguide transmission when GST is in the crystalline state. Further, by increasing the optical probe power, the readout state can be transformed from non‐volatile to volatile (Figure 12i), which provides a promising platform for all‐optical data storage and computation. The phase change material GST has made a great process in the past few years, and quite several reports have proved its fast speed and superior stability in photoelectric computing. At present, some functionalities and stacked metallic nanorods GST have exhibited unique tunable optical properties and have already been applied in nanophotonic components.^[^ ^252^, ^253^ ^]^ In short, functionalizing and reducing the dimension of the GST is a new pathway to broaden the application scenarios of the photonic memristive and memristive‐like devices.

As the prevalent material, polymers have been widely applied in various devices owing to their high feasibility, low toxicity, and flexibility.^[^ ^254^, ^255^, ^256^ ^]^ Regarding the memristor, the polymers can be acted as a blocking layer and floating layer, contributing to the resistive switching behaviors.^[^ ^257^, ^258^ ^]^ However, given the low light absorption and light insensitivity, few reports mention the application of polymer in photonic memristive devices. To apply the advantages of polymer to photonic memristive devices, it is necessary to develop polymer‐based photonic memristive devices with photo‐response. Kemp et al. enable the optically active azobenzene polymer PDR1A onto a vertical array of ZnO nanorods, and, construct the polymer‐based photonic memristive devices with superior light‐induced ON/OFF ratio.^[^ ^124^ ^]^ The PDR1A undergoes a *trans*–*cis* photochemical isomerization upon optical excitation, resulting in the transformations between HRS to LRS. Zhou et al. propose a UV damage sensing nociceptive photonic device, of which structure is exhibited in Figure 12j.^[^ ^238^ ^]^ A film of PMMA doped with Azobenzene‐functionalized gold nanoparticles (Azo‐Au NPs) acts as the resistive switching layer. Figure 12k shows the schematic diagram of the Azo‐Au NPs and molecular view of the Azo ligand under visible and UV light. Thereinto, the photoisomerization between two structural conformations in an azobenzene‐containing component can be triggered when the optical irradiation is applied to the device, and the material's properties of the active film change simultaneously, resulting in the decrease of the SET voltage of the resistive switching behavior. As the detailed molecular transformation of the Azo ligand (Figure 12l), the device shows a trans‐state in the initial state (Figure 12l‐I) and exhibits the high SET voltage in resistive switching. While it is converted to the cis state by the triggering of the UV irradiation (Figure 12l‐II), which decreases the SET voltage from 2.8 to 1.5 V.

Based on the flexibility of the materials, we do not strictly classify and define when describing the material system. Actually, in light of the limitation in the current preparation technology and theoretical knowledge, no material can effectively realize the large‐scale commercial use of photonic memristive and memristive‐like device although there are so kinds of materials to construct them. Therefore, as the core part of the photonic memristive and memristive‐like device, the selection and preparation of active layer materials require further research and experiments by the majority of researchers.

## 4 Applications of Photonic Memristive and Memristive‐Like Devices

As we know, the traditional electronic memristive devices have developed a variety of application scenarios in the past ten years, such as the traditional RRAM,^[^ ^133^, ^259^ ^]^ logic operations integrated with memory,^[^ ^172^, ^174^ ^]^ and the recently remarkable biological synapses.^[^ ^260^, ^261^ ^]^ However, the applications of the traditional electronic memristive devices have been impeded due to the single function, weak expansibility, device vulnerability, and narrow bandwidth.^[^ ^248^, ^249^, ^250^, ^251^, ^252^, ^253^, ^254^, ^255^, ^256^, ^257^, ^258^, ^259^, ^260^, ^261^, ^262^, ^263^ ^]^ In order to satisfy various kinds of applications, the research of novel memristive devices is imminent. Recently, with the merits of non‐contact operation, non‐destructive properties, and low power consumption, photonic memristive devices have attracted great attention, which enables them to better realize the function of traditional electrically controlled memristive devices. After coupling with light, the memristive devices can adapt to many demanding applications, such as artificial visual neural networks,^[^ ^65^ ^]^ optical encryption communication,^[^ ^145^ ^]^ image processing,^[^ ^64^ ^]^ and visual memory systems.^[^ ^69^ ^]^ In the following, we will introduce the application scenarios and advantages of the photonic memristive and memristive‐like devices to its future applications.

### 4.1 Photo‐Induced Multilevel RRAM

Until now, RRAM is the most widely used and closest to the practical commercial applications of memristive devices. However, the advent of the big data era makes it difficult for the traditional RRAM to meet the requirements of high‐density and large‐scale storage systems. To increase the storage density, the multilevel RRAM can be achieved by tuning the compliance current or the sweeping voltage in a unit device. Compared with the traditional electronic RRAM, photonic memristive devices can achieve the transformation between HRS and LRS under the effect of electric, light, and their combined modulation. This property allows the device can be transformed among at least three states, leading to the multilevel resistive switching behaviors. Because of the superiorities of the light, such as the adjustable intensity, variable wavelengths, and low power consumption, photonic memristive devices have superior practicality in realizing multilevel resistive switching behaviors.^[^ ^208^, ^212^ ^]^ Therefore, the concept of photo‐induced multilevel RRAM came into being.

**Figure 13:**
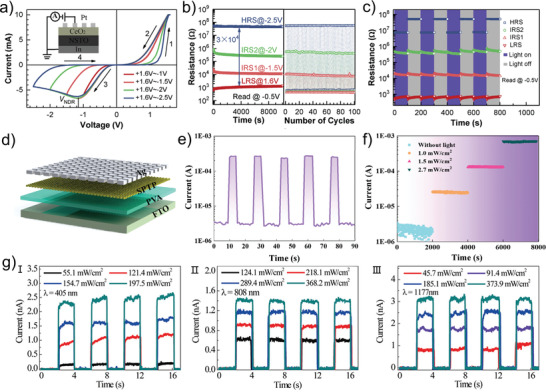
a) The *I*–*V* characteristics of the Pt/CeO_2_/Nb: SrTiO_3_/In RRAM. b) The retention and endurance characteristics of the device at LRS, IRS1, IRS2, and HRS. c) The retention properties of the device at LRS, IRS1, IRS2, and HRS in dark and under illumination. d) Schematic of the Ag/SPTP/PVA/FTO structured multifunctional photonic RRAM. e) Current–time response of HRS under UV light (1.5 mW cm^−2^) and dark conditions. f) Multilevel resistance states achieved by UV modulation with light intensities of 0, 1, 1.5, and 2.7 mW cm^−2^. g) Photoresponse characteristics of the PbS QDs‐based device under various laser irradiation. Light wavelengths: I) 405 nm, II) 808 nm, III) 1177 nm at various power densities. (a–c) Reproduced with permission.^[^ ^264^ ^]^ Copyright 2018, Elsevier B.V. (d–f) Reproduced with permission.^[^ ^258^ ^]^ Copyright 2021, Wiley‐VCH. (g) Reproduced with permission.^[^ ^106^ ^]^ Copyright 2020, Royal Society of Chemistry.

Li et al. present a photo‐induced multilevel RRAM based on Pt/CeO_2_/Nb: SrTiO_3_/In, of which structure and *I*–*V* behaviors are shown in **Figure** **13**a.^[^ ^264^ ^]^ This device can be transformed among four states under the electric field, including the LRS, Intermediate state1 (IRS1), IRS2, and HRS (Figure 13b). When the device is exposed to 405 nm light, the HRS increases significantly, while the other states are almost invariant, as shown in Figure 13c. This device only adds one state under a specific wavelength of light, which is not reflecting the low power consumption and the adjustable intensity superiorities. Inspired by the adjustability of the light intensity, Li et al. propose a novel multifunctional photonic RRAM based on the surface‐plasma‐treated inorganic halide perovskite (SPTP), of which structure is exhibited in Figure 13d.^[^ ^258^ ^]^ Figure 13e shows that the SPTP‐based RRAM takes on the HRS in the initial state, then it is tuned to LRS under the UV modulation. By varying the applied intensity of the UV light (0, 1, 1.5, and 2.7 mW cm^−2^), five resistance states are achieved, and the conductance of the device increases with the increasing light intensity, which is exhibited in Figure 13f. It is contemplated that, limited by the materials, the transformation of multilevel states is dependent on the light intensity in most photo‐induced multilevel RRAMs. Lately, Zhang et al. construct the light intensity and light wavelength sensitive photo‐induced multilevel RRAM, which is based on PbS QDs/PMMA composite film.^[^ ^106^ ^]^ As shown in Figure 13g, the photocurrent of the device is increasing with the increase of power density for illumination at the same wavelength. Furthermore, it also can be observed that 12 states can be obtained by adjusting the light intensity under different wavelengths (Figure 13g‐I: 405 nm, Figure 13g‐II: 808 nm, Figure 13g‐III: 1177 nm, respectively). More remarkable, the device in this work has the most states to date. Typically, the introduction of illumination broadens the idea of constructing a novel photo‐induced multilevel RRAM and provides strong support for further improving the storage density, reducing the electrical damage and power consumption of devices.

### 4.2 Photoelectric Logic Operations

**Figure 14:**
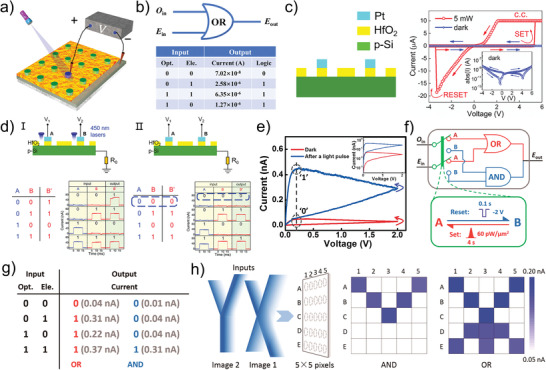
a) Schematic diagram of the photoelectric logic RRAM with the structure of TiN/BiVO_4_/FTO/Glass. b) Logic gate with OR operation and the output table. c) Planar implementation of the Pt/HfO_2_/p‐Si devices and the *I*–*V* characteristics under illumination with 450 nm light. d) Light‐controlled IMP operations I) with and II) without light illumination. e) Resistive switching of the device before and after the light at the positive voltage sweep. Inset: the log‐scale *I*–*V* curve. f) Schematic diagram of the “AND” and “OR” logic operation switching of the ITO/CeO_2−_ *_x_*/AlO*_y_*/Al memlogic devices. g) Truth table and output current values of the memlogic “AND” and “OR.” h) Proof‐of‐concept demonstration of image recognizing and memorizing. (a, b) Reproduced with permission.^[^ ^142^ ^]^ Copyright 2018, American Institute of Physics. (c, d) Reproduced with permission.^[^ ^271^ ^]^ Copyright 2018, American Institute of Physics. (e–h) Reproduced with permission.^[^ ^7^ ^]^ Copyright 2017, American Chemical Society.

With the improvement of the semiconductor device performance and integration, the traditional von Neumann architecture has encountered a bottleneck owing to the transmission speed and bandwidth limitation between center processing unit and memory.^[^ ^265^, ^266^, ^267^, ^268^ ^]^ Compared with the conventional computing based on the von Neumann architecture, memristive devices, which are able to store and process information in a unit cell, have become one of the promising candidates for the next‐generation memory.^[^ ^269^, ^270^ ^]^ Furthermore, to increase the parallelism of the operations and reduce the power consumption, light as the external stimulus other than voltage is introduced, which can realize the photoelectric logic operations. In a typical photoelectric logic device, the output of the logical operations is dependent on the interaction of voltage and light. For example, as shown in **Figure** **14**a, the TiN/BiVO_4_/FTO device proposed by Yan et al. realizes the logic “OR” by applying electricity and light.^[^ ^142^ ^]^ Thereinto, voltage and light signals act as two independent inputs. In the initial state, the state of the device exhibits HRS due to the “0” input of voltage and light. While if any input becomes “1,” the final output result is “1,” which realizes the logic “OR,” as shown in Figure 14b. In particular, compared with the simple logic “AND,” “OR,” and “NOR,” the logic “IMP” and “false” are the logical basis for a complete computing system. Up to now, the logic “IMP” and “false” have been achieved by applying the photoelectric logic operations. Ordinarily, Xia et al. propose a p‐Si/HfO_2_ heterojunction photonic memristive devices, of which structure and the *I*–*V* curve are shown in Figure 14c.^[^ ^271^ ^]^ Firstly, the logic “false” is realized by applying the *V*_RESET_ voltage on the top electrode. When the *V*_RESET_ comes, the state of the device is reset to “0” regardless of the previous state. In addition, as shown in Figure 14d‐I, a resistance *R*_0_ is connected in a series of two devices (A and B) to realize the logic “IMP.” The voltage *V*_1_ and *V*_2_, applied on devices A and B, respectively, should satisfy two conditions, that are, *V*_2_ > *V*_set_ > *V*_1_ and *V*_2_−*V*_1_ < *V*_set_, which makes that the individual *V*_2_ stimulus can program all devices in logic “1” and the individual *V*_1_ stimulus retain the origin state. When the light is on, the state of B is dependent on the A. If the state of A is “0,” the B is always “1,” while B can retain the original state when the A is “1,” which realizes the logic “IMP.” As shown in Figure 14d‐II, based on the basic logic “IMP,” the logic “OR” and “NAND” can be realized by tuning the sequence of devices or voltage, indicating the potential in combinational logic operations.

In recent years, with the merits of high reprogram ability, reconfigurable logic circuits have been considered as an effective approach to enhance computing performance in the post‐Moore era. Memristive devices, as the most potential reconfigurable logic device in next‐generation memory that can store the assigned function and output data, have been attracted attention for realizing reconfigurable logic circuits. Li et al. have done a lot of research on photoelectric reconfigurable logic devices. The most representative work is the ITO/CeO_2‐_ *_x_*/AlO*_y_*/Al‐based device published in 2017, of which structure is shown in Figure 7e.^[^ ^7^ ^]^ This device can realize the transformation between logic “AND” and “OR” by applying light to the device, illustrating the reconfigurable potential. As shown in Figure 14e, the device exhibits resistive switching behaviors in both dark and light, and the conductance of the device is significantly improved after a light pulse. Subsequently, a memlogic circuit is constructed to realize the reconfigurable operation (Figure 14f), in which the voltage (*E*_in_) and input (*O*_in_) act as two inputs. As shown in Figure 14g, the device can realize the logic “AND” in the dark conditions, while the logic “OR” can be achieved in the light conditions. In addition, the logic “NOT” can be realized by pre‐programming the device state and introducing the *V*_RESET_. Especially, the potential of this device in image processing is investigated by designing a 5 × 5 array. As shown in Figure 14h, the image processing function of “same finder and all finder” is realized by arraying the logic “AND” and “OR,” which paves the way for the parallel operations for data storage and processing in a unit memory and reconfigurable logic operations. In summary, the photonic memristive device is an ideal device to construct a new type of memory with the logic operation and break through the traditional Von Neumann bottleneck.

### 4.3 Photonic Memristive and Memristive‐Like Synapses for Neuromorphic Computing

It is well known that biological nervous systems exhibit the merits of compact, fault‐tolerant, and efficient in complex perception, learning, and memory.^[^ ^13^, ^61^, ^95^, ^262^, ^263^ ^]^ A nervous system composed of 10^15^ synapses interconnected is the basis for the human brain to process and store information. Among them, as a unit capable of independent memory and storage, synapses can process large amounts of information in parallel, and their power consumption for each event is about 100 fJ. Inspired by these powerful signal processing abilities of the human brain, synapse‐like artificial devices have been proposed to mimic the processing and transmission of signals in a human brain.^[^ ^19^, ^53^, ^61^ ^]^ The memristive devices are regarded as the most candidate device for constructing the artificial network owing to the similarity to the structure and performance of the biological synapse. Unfortunately, the pure electronic memristive synapse has a bottleneck due to the high power consumption, low conductivity variation, and low linearity. Compared with the pure electronic synapse, the photonic memristive and memristive‐like synapses have lower power consumption, faster processing speed, and larger bandwidth, which are beneficial to the performance in simulating the EPSC, short‐term plasticity (STP), long‐term plasticity (LTP), PPD, PPF, and SRDP.^[^ ^47^, ^64^, ^65^ ^]^

**Figure 15:**
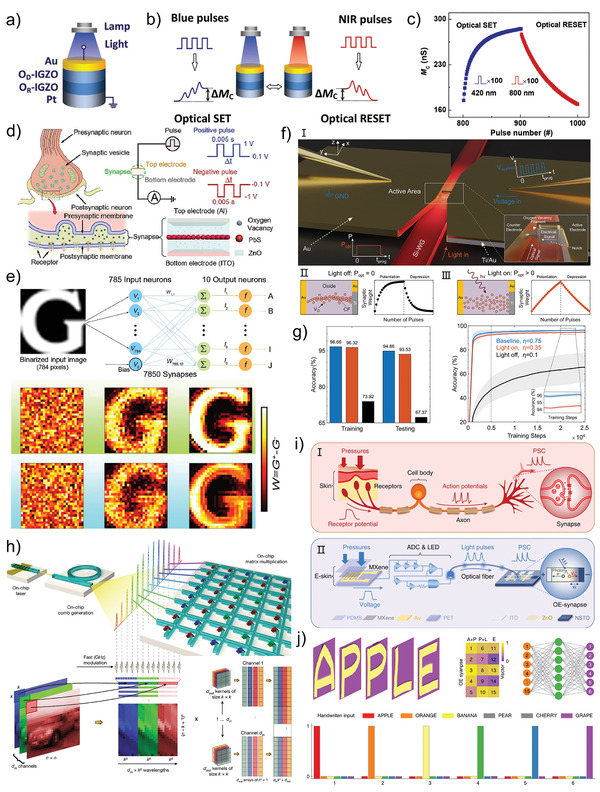
a) Schematic diagram of the Au/OD‐IGZO/OR‐IGZO/Pt photonic memristive device. b) Measurement schematic and the optical pulse waveform of the AOC photonic memristive device. c) Reversible regulation of the conductance under all‐optically mode (Blue pulses: SET, Red pulses: RESET). d) Schematic demonstration of biological synapse structure and the illustration of the ZnO/PbS artificial synapse. e) Schematic illustration of simulated ANN by 7850 synaptic weights with 785 input neurons and 10 output neurons and the mapping images of 784 synaptic weights connected to output letter “g” in two modes. f) Operating principle of the nanoscale photonic memristive‐like synapse, Ι) Schematic illustration of a planar plasmonic Au‐HfO_2_‐Ti/Au slot waveguide with a notch. II) A nonlinear, nonsymmetric response of the memristor conductance to electrical pulses follows. III) More linear and more symmetric of the memristor conductance to light pulses follows. g) Maximum training (left) and testing (right) recognition accuracies with (red) and without (black) light as well as with ideal memristors (baseline case, blue) and the accuracy of the handwritten digit recognition over 15 epochs as a function of the training steps. h) Photonic in‐memory computing using a photonic‐chip‐based microcomb and PCMs. i) Schematic diagram of the biological afferent nerve systems and the artificial afferent nerve systems. j) Classification of handwritten words. (a–c) Reproduced with permission.^[^ ^269^ ^]^ Copyright 2020, Springer Nature. (d, e) Reproduced with permission.^[^ ^95^ ^]^ Copyright 2018, Wiley‐VCH. (f, g) Reproduced with permission.^[^ ^276^ ^]^ Copyright 2021, American Chemical Society. (h) Reproduced with permission.^[^ ^282^ ^]^ Copyright 2021, Springer Nature. (i, j) Reproduced with permission.^[^ ^287^ ^]^ Copyright 2020, Springer Nature.

Zhou et al. propose a CsPbBr_3_ QDs‐based photonic memristive synapse, which can realize the performance of photonic potentiation and electrical habituation.^[^ ^95^ ^]^ The transition from STP to LTP and the PPD, PPF, SRDP properties are achieved based on the adjustable light intensity and irradiation time. Worth noting that the energy consumption for a single pulse event is 1.4 × 10^−9^ J, which is defined as *I*_peak_ × *t* × *V*, where *I*_peak_, *t*, and *V* are peak values of the EPSC, pulse duration time, and pulse voltage, respectively. The power consumption of the proposed photonic memristive synapses is significantly lower than the pure electronic memristive synapses, which is close to the biological synapses. Furthermore, the photonic memristive synapses also exhibit larger conductance variation compared with the pure electronic memristive synapses. In the work mentioned above, a negative voltage pulse is necessary to erase the memorized light information, which results in extra power loss and signal transmission delay. While the key to conquering these issues is to realize a light write and erase operation, that is the all‐optical mode.^[^ ^272^, ^273^, ^274^, ^275^ ^]^ In terms of these issues, Zhuge et al. demonstrate an all‐optically controlled (AOC) analog memristive device with a heterostructure of Au/oxygen‐deficient IGZO (OD‐IGZO)/oxygen‐rich IGZO (OR‐IGZO)/Pt (**Figure** **15**a).^[^ ^272^ ^]^ The performance of the AOC photonic memristive device is investigated under the consecutive Blue and NIR light pulses, as the measurement diagram shown in Figure 15b. Consecutive blue pulses excite an increase in the photocurrent, meaning an optical SET process. And a strong decrease in the current occurs when the consecutive NIR pulses are applied, indicating the optical RESET process. Figure 15c exhibits the achieved all‐optically mode in the AOC photonic memristive device. It can be observed that the conductance can be reversible regulated even with only the optical signals applied. In this case, the RESET process consumes 2.4 × 10^−10^ J in a single AOC photonic memristive device with a 100 µm × 100 µm cell area, which is significantly lower than that of the electric erase case. In addition, the all‐optically photonic memristive synapses exhibit superior potential in neuromorphic computing due to the fast response speed, wide response wavelength, and low noise factor. Zhou et al. have also constructed a full‐photo modulated hetero‐structure for neuromorphic computing, of which structure is based on Al/ZnO/PbS/ZnO/ITO (Figure 15d).^[^ ^64^ ^]^ Thereinto, the potentiation process is realized by implementing UV light to the device, while the NIR light leads to the depression process. The light‐induced PPD/PPF properties are achieved by applying various frequency UV/NIR light pulses. Mentioned that the energy consumption in fully photon modulation of this work is calculated to 4 × 10^−12^ J with 20 µm × 20 µm cell area. This device exhibits promising potential in image recognition because of the superior full‐photo modulated and low noise behaviors. As shown in Figure 15e, an image is divided into 28 × 28 = 784 patches, where every patch represents a pixel connected to the 784 neurons. The input layer is composed of 784 neurons with one bias neuron, nine interlayer neurons, and 10 output neurons corresponding to 10 letters from “a” to “j.” All neural network layers constitute the artifical neural networks (ANN) to realize neuromorphic calculations, and the group draws the mapping image of the output letter “g” in two modes after training. Compared with the untrained, the recognition rate of the image after 1000 iterations of training can reach 67 ± 6%. On this basis, the team also performs similar simulations in up‐conversion particle photonic memristive synapses. After the same 1000 training, the device exhibits up to 70% recognition accuracy. Even though coupled light increases responsivity without affecting the linearity of the conductance change in many oxide‐stacked photonic memristive devices, the inherent random ion migration and diffusion behaviors cause nonidealities such as abrupt switching between their LRS and HRS, low retention times, and device‐to‐device variability. Further, a crossbar array of photonic memristive devices with poor linearity and stability is inaccurate and incapable in neuromorphic computing. Aiming at these issues, a nanoscale photonic memristive‐like synapse is designed by Emboras et al., which consists of a planar silicon photonic waveguide located below a plasmonic slot made of an Au‐HfO_2_‐Ti/Au VCM memristive stack, as shown in Figure 15f‐I.^[^ ^276^ ^]^ When the light is injected into the plasmonic cavity, the lattice temperature is heated due to the confined optical energy, which boosts the generation rate of oxygen vacancies in the active area, while simultaneously enhancing their diffusion coefficient. In addition, benefiting from the enhanced diffusion coefficient, the nonlinearity as well as the asymmetry of the long‐term potentiation/depression is strongly reduced compared with the electric modulated mode, as shown in Figure 15f‐II,III. Hence, the proposed device exhibits better compatibility with deep learning applications through light irradiation. As shown in Figure 15g, by inserting the measured conductance modulation behavior into a neural network simulator, the light‐enhanced recognition accuracy of a deep neural network is proved to be 93.53%, similar to ideally performing memristors (94.86%).

Despite the numerous photonic memristive devices proposed in recent years, performing computational tasks in a memristor‐chip that implements neural networks computing is extremely challenging due to the complicated parallel data processing method, inaccurate optical phase control, and difficult integrated approach. First, implementing matrix‐vector multiplication at the memristor hardware circuit level is required for all‐photonic chip‐scale information processing, since it is one of the essential processing processes in neural networks computing.^[^ ^277^, ^278^, ^279^ ^]^ In 2019, Bhaskaran et al. first proposed a photonic computational memory based on phase‐change materials GST in combination with Si_3_N_4_/SiO_2_ photonic waveguides.^[^ ^280^ ^]^ The proposed device exhibits memristive‐like properties owing to the distinctive pinched hysteresis characteristic between reflected and incident light power and it can perform a direct multiplication of scalar numbers using distinct interaction of two light pulses, of which results provide a foundation for collocated memory and processing on a photonic platform. Then, an all‐optical spiking neuron circuit, constructed by GST‐based all‐photonic memristive‐like synapses, is proposed to implement circuit‐level matrix‐vector multiplication. The circuit consists of four neurons and sixty synapses and further uses a wavelength division multiplexing (WDM) layered architecture to improve optical data processing speed.^[^ ^281^ ^]^ A prototypical artificial intelligence task of pattern recognition is realized in both a supervised and unsupervised manner using all‐optical spiking, bridging the gap between hardware neural networks and artificial photonic memristive synapses and providing a new pathway for subsequent direct processing of optical telecommunication and visual data. In the recent study by Bhaskaran et al., an optical analog of an application‐specific integrated circuit named integrated photonic tensor core or photonic memristive‐like device is realized aiming to perform convolutional processing.^[^ ^282^ ^]^ In this work, using CMOS‐compatible phase‐change materials and implementing WDM in conjunction with multichannel sources (that is, optical frequency combs), several key issues in the hindering large‐scale fabrication and application of photonic devices, such as compatibility with CMOS process, utilization of broadband multi‐channel laser source, and precise control of the optical phase, are effectively solved. As shown in Figure 15h, photonic in‐memory computing is constructed by stacking the kernel matrices into the columns of the final filter matrix, and the single convolution operation involves (*n* − *k* + 1)^2^ MVM operations between the filter matrix and the input vectors of dimension *k* ^2^ × *d*_in_, further achieving the image convolution processing. A parallel, fast, and efficient computational hardware neural network is performed in the photonic tensor core, providing a robust hardware platform for data‐heavy AI applications including autonomous driving, live video processing, and next‐generation cloud computing services. Currently, such photonic neurosynaptic networks constructed by the phase‐changes materials with a photonic waveguide have been demonstrated a unique role in constructing the photonic platform towards commercialization.

Recently, integration and cooperation of tactile sensory, neurons, and synapses in the bionic system are pursued to process the tactile information. By detecting the external stimuli produced by the environmental variation, the action potential (spikes) is transferred to electrical signals by the tactile sensory, and finally, process the detected information with neural coding and learning. Moreover, through the synergistic action of the tactile sensory and synapse, the bionic system can be applied in image recognition,^[^ ^283^, ^284^ ^]^ speech recognition,^[^ ^285^, ^286^ ^]^ and material identification.^[^ ^287^, ^288^, ^289^ ^]^ However, the electric‐modulated operation and contact integration in the traditional electric‐based bionic system bring high power consumption, high noise, and vulnerability to the device. Thus, the mode of communication between the tactile sensory and memristor has transferred from electrical to optical conduction. An photonic spiking afferent nerve has been proposed by Tan et al., which is constructed by a MXene‐based tactile sensory and ITO/ZnO/NSTO photonic memristive device.^[^ ^287^ ^]^ Figure 15i‐I depicts the schematic diagram of the biological afferent nerve. Thereinto, the pressure signals are perceived by the skin and converted to the action potentials by the neuron, finally inducing EPSCs in the synapse. Inspired by the biological afferent nerve, an artificial afferent nerve system, consisting of a ring oscillator, an edge detector, an LED, and a photonic memristive device, is constructed to emulate the biological behaviors, as shown in Figure 15i‐II. The perceived pressure signals are converted to the light signals through the edge detector and LED, and received by the photonic memristive device with neural coding and learning. Utilizing the pressure‐dependent multiple coding principles, the artificial afferent nerve system can realize the in‐memory computation of sensory data. For instance, the Morse code reader function can be achieved by varying the touch time in the tactile sensory, which results from the longer spiking time in the PSCs under longer touching conditions. And, the classification of handwritten words is achieved by discriminating the various tactile information. As shown in Figure 15j, the handwritten words “ORANGE,” “BANANA,” “PEAR,” “CHERRY,” and “GRAPE” can be represented by 15D vectors, and an artificial neural network is constructed to classification. All words are recognized successfully after only four training cycles, as each output neuron responds to only one input word. To summarize, the establishment of this photonic memristive synapse not only offers enormous promise for the development of a new computer system but also introduces a novel concept for the implementation of neural network computing.

### 4.4 Photonic Memristive and Memristive‐Like Synapses for Artificial Visual System

Photonic memristive synapses, possessing the advantages of superior photocurrent and persistent photoconductivity effect, have been applied in the artificial visual system in recent years.^[^ ^290^, ^291^, ^292^, ^293^, ^294^, ^295^, ^296^, ^297^ ^]^ As we know, human visual memory is the most intuitive and effective way of memory in the human brain and can be considered as the start of human memory, and nearly 80% of the information is obtained through the eyes. With the fast progress of modern society, artificial intelligence robots and light‐driven neurorobotic are urgently desired to replace human vision in scientific,^[^ ^295^, ^296^, ^297^ ^]^ industrial,^[^ ^298^, ^299^, ^300^ ^]^ and military scenarios^[^ ^301^, ^302^, ^303^ ^]^ to work under complicated situations. Thus, emulating the visual functions is the essential part to achieve the electronic eyes and advanced robot systems.^[^ ^304^, ^305^, ^306^ ^]^ Photonic memristive and memristive‐like synapses give a novel pathway to emulate the visual memory functions in view of its perception and memory property to light.

**Figure 16:**
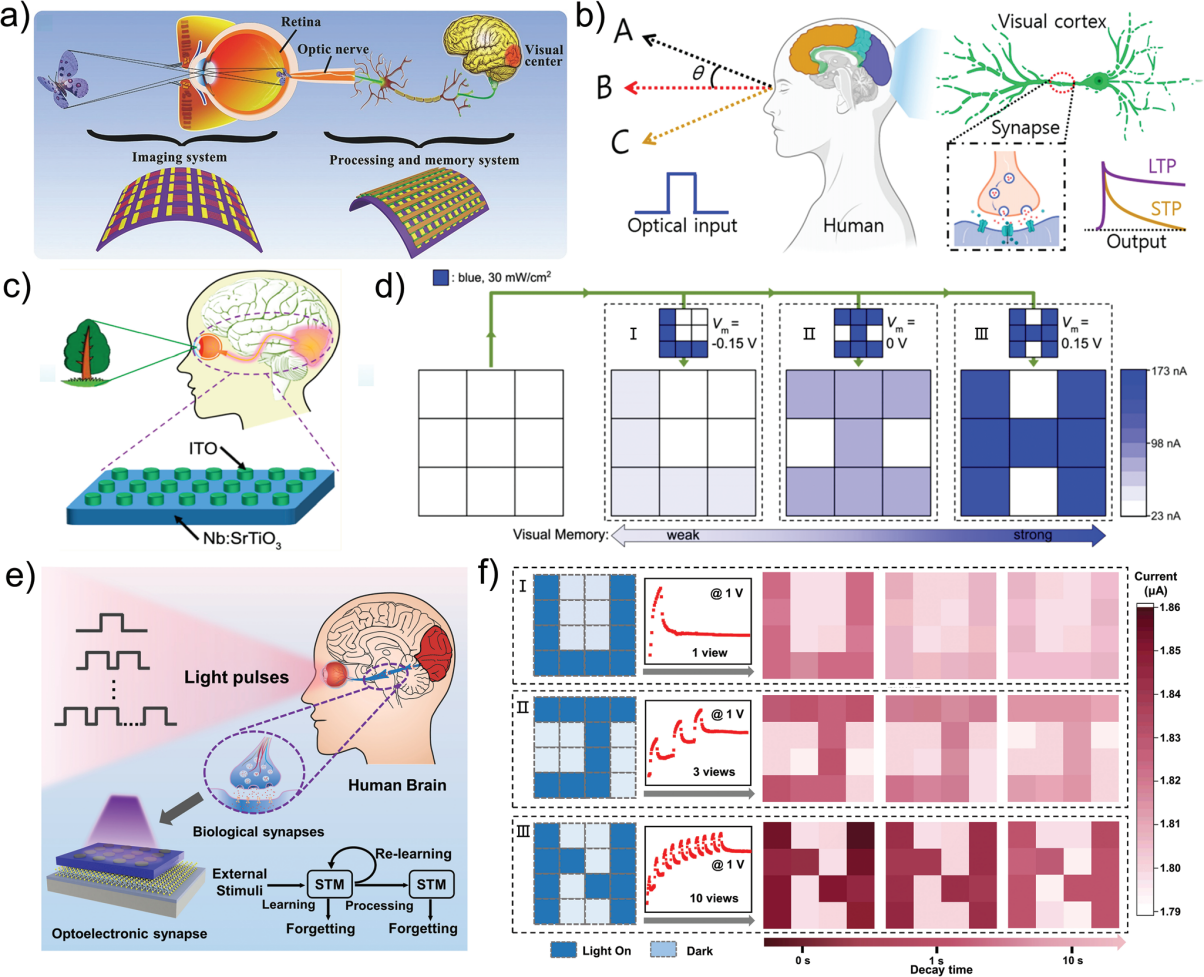
a) Schematic diagrams of the human visual system when a butterfly is observed by eyes. b) Schematic of the photonic‐electronic‐coupled neuromorphic angular visual system. c) Schematic of the interest‐modulated human visual memories based on the ITO/Nb:SrTiO_3_ heterojunction. d) Mimicry of interest‐modulated human visual memory with I) low interest represented by *V*_m_ = −0.15 V, II) intermediate interest represented by *V*_m_ = 0 V, and III) high interest represented by *V*_m_ = 0.15 V. e) Schematic of the light pulse numbers‐dependent photonic memristive synapses based on the TiN*_x_* O_2‐_ *_x_*/MoS_2_ heterojunction. f) Conductance response image mapping after I) 1 light pulse, II) 3 light pulses, and III) 10 light pulses. (a) Reproduced with permission.^[^ ^289^ ^]^ Copyright 2018, Wiley‐VCH. (b) Reproduced with permission.^[^ ^149^ ^]^ Copyright 2020, American Chemical Society. (c, d) Reproduced with permission.^[^ ^65^ ^]^ Copyright 2019, American Chemical Society. (e, f) Reproduced with permission.^[^ ^72^ ^]^ Copyright 2021, Wiley‐VCH.

Many effective approaches and methods for constructing photonic memristive and memristive‐like synapses have been reported in recent years. Shen et al. propose a flexible artificial visual memory system based on a UV‐motivated memristive device.^[^ ^289^ ^]^ As shown in **Figure** **16**a, the human visual memory system can be divided into two parts, including the retina with perception and the optic nerve network with memory. Thus, the integration of UV image sensors (In_2_O_3_ semiconductor micrometer‐sized wires) and memristive device (Ni/Al_2_O_3_/Au) is emulated the perception and memory functions, respectively. This proposed architecture provides a novel pathway to integrate functional sensors and memory devices for the imitation of human echoic memory and haptic memory. It should be emphasized that the device structure in which sensing and memory are separated is unfavorable to improving device integration and reducing power consumption. Memorizing and detecting images in a unit device is an effective method to solve these issues. Seo et al. provide a simple approach to detect and memory the visual image in a unit device, which is constructed by the Ag NWs/TiO_2_ Schottky optoelectronic‐coupled architecture.^[^ ^149^ ^]^ Benefiting from the light sensitivity of the TiO_2_, the proposed device does not need extra photodetectors to sense light signals, and the resistive switching behaviors of the TiO_2_ give rise to the memory function, integrating the perception and memory functions. In especially, in light of the different absorbance light under various off‐normal incident angles of the present device, the Ag NWs/TiO_2_ device can detect the broad angular light information, as shown in Figure 16b. Thus, this work provides a novel pathway for emulating broad angular visual perception with a simplified circuit. Li et al. propose a simple artificial photonic memristive synapse based on the ITO/Nb:SrTiO_3_ heterojunction, of which structure is exhibited in Figure 16c.^[^ ^65^ ^]^ The proposed photonic memristive synapse shows self‐adaptive optical signal detecting, integrated visual information processing, and memorizing functions in a unit cell, and the voltage modulated photo‐plasticity is realized by tuning the reading voltage, which means that the proposed photonic memristive synapse is suitable for the mimicry of interest‐modulated human visual memories. As shown in Figure 16d, the photo‐response of the device increases with the reading voltage. The larger reading voltage can represent the image with higher interest, and the lower reading voltage shows the lower interest in the image. The voltage modulated method is an effective method to tune the photo‐response and the photo‐plasticity, but it has significantly enhanced the operation complexity. To conquer these problems, Li et al. proposed a TiN*_x_* O_2‐_ *_x_*/MoS_2_ heterojunction‐based photonic memristive synapse, which is tuned by the light pulse numbers (Figure 16e).^[^ ^72^ ^]^ To be more specific, the conductance of the photonic memristive device can be modulated by adjusting the numbers of UV‐light illumination to emulate the human visual system. In this pathway, the numbers of light stimuli applied to visual neurons are emulated by the numbers of UV‐light illumination, which realistically emulates the biological behaviors and simplifies the complexity of the conductance regulation. Under the different applied light pulses (1, 3, and 10), the image mappings “U” (Figure 16f‐I), “J” (Figure 16f‐II), and “N” (Figure 16f‐III) are predefined in the 4 × 4 array, respectively. With the applied light pulses increase, the image becomes clearer and the duration increases, indicating the enhancement of the visual memory. The proposed device shows promising potential in the realization of artificial neuromorphic computing and artificial visual system.

The application of photonic memristive synapses in visual memory systems has developed rapidly in recent years. As mentioned above, a variety of simulation methods have been used to emulate the biological eye, including voltage modulation, light wavelength modulation, light pulse numbers modulation, and angle modulation. These simulation methods greatly promote the construction of electronic eyes. However, the biological visual system is extremely complex and advanced. Through information interaction between the retina and the human brain, the biological eye can capture and process the specified multi‐dimensional light information in the complex external environment, and then store the effective information in the human brain for a short time or a long time. At this stage, photonic memristive synapses cannot realize the complex processing of the multi‐dimensional optical signal, so there is still a long way to go from the actual construction of the electronic eyes and advanced robot systems.

## 5 Conclusion and Outlook

Being capable of handling both electrical signals and light, the emerging photonic memristive and memristive‐like devices may open up a new era for computational systems owing to its high efficiency, low‐power consumption, low‐crosstalk, noncontact, and nondestructiveness properties. In this review, we focus on the photonic memristive and memristive‐like devices in the aspect of mechanisms, materials, and applications. Through analyzing recent advances in photonic memristive and memristive‐like devices, the state‐of‐art, the strength, and the weaknesses of the them are summarized and discussed in detail. Fully understanding and explaining the working mechanism is crucial to its development, thus the photo‐induced resistive switching mechanisms are elaborated at first. Subsequently, we focus on the materials to construct the photonic memristive and memristive‐like devices and illustrate the advantages and disadvantages in combination with several key optical and electrical properties, such as light absorption coefficient, photosensitive wavelength, and photochemical stability. Ultimately, we summarize the potential application of the photonic memristive and memristive‐like devices in various aspects such as multilevel RRAM, photoelectric logic operations, neuromorphic computing, and artificial visual system.

So far, the research on photonic memristive and memristive‐like devices has made great progress in the past few years, although it is still in its infancy compared with the traditional electronic memristive devices. Looking ahead, the practical applications of photonic memristive and memristive‐like devices still have a long way to go, and various existing problems need to be solved and features need to be discovered, especially in the following aspects:

(1) Based on the traditional electrical resistive switching mechanisms, the photo‐induced resistive switching mechanisms are divided into photo‐generated carriers, photo‐mediated interface barrier, photo‐induced formation/annihilation of conductive filaments, and photochemical reaction process. Owing to the different conductance change regulations for the various mechanisms, selecting the suitable mechanism is the key factor for the practical application in the RRAM, artificial synapse, and visual network. Although various photo‐induced resistive switching mechanisms are proposed and adopted, many stable and useful mechanisms remain to be discovered limited by characterization methods and information occlusion between disciplines. Taking advantage of the latest characterization facilities and the novel photosensitive materials is an effective way to improve the existing photo‐induced resistive switching mechanisms.
(2) Selecting suitable photosensitive materials is the core of enhancing the photoelectric resistive switching performance during the construction of the active layer. Until now, various photosensitive materials, including metal oxides, 2D materials, quantum dots, perovskites, etc., have been proposed to construct the photonic memristive and memristive‐like devices. The preparation and utilization of these materials is an important component to improving and enhancing the photonic memristive and memristive‐like devices material system. However, even if the above materials show excellent optical properties, many key problems in the synthesis and preparation process are still to be solved, such as the homogeneity, large‐area preparation, low detection rate, and environmental stability. At this stage, further research on the advantages and disadvantages of each material applied to the appropriate application is an effective way to solve the above problems. Besides, searching for more universal materials, which possess stable and efficient photoelectric performance and the ability for large‐scale production, is the key to achieving photonic memristive and memristive‐like devices in RRAM, logic computing, and visual memory system in the future.
(3) With the improvement of mechanisms and materials, photonic memristive and memristive‐like devices have been applied in various fields, such as multilevel RRAM, photoelectric logic operations, neuromorphic computing, artificial afferent nerve system, and artificial visual system. At this stage, the application of multilevel RRAM and photoelectric logic operation has been relatively mature. The high‐power consumption, high crosstalk, and high loss characteristics of traditional memristive devices have been overcome, opening up a new avenue for high‐density storage and in‐memory computing. Regarding the applications in neuromorphic computing, photonic memristive and memristive‐like devices‐based neural networks for image processing as well as pattern recognition are widely and effectively implemented, and the operating modes of these devices are typically based on optical‐write and electrical erase. Nevertheless, to optimize the calculation speed and decrease power consumption, an ideal photonic memristive or memristive‐like device that can be modulated in an all‐optically mode is needed. There have been a few reports on all‐optically tunable photonic memristive and memristive‐like devices so far, but their implementation remains a challenge. In addition, due to the complex structure and functions of the biological eye, such as the multi‐dimensional response to the external natural light signal and the unique flexibility of the eyeball, the application of the artificial visual system is still in its infancy. Photonic memristive and memristive‐like synapses cannot realize the complex processing of the multi‐dimensional optical signal, so there is still a long way to go from the actual construction of the electronic eyes and advanced robot systems. Facing these issues, collaboration in various fields such as neuroscience, solid‐state electronic science, and computer science is needed to explore the specific connection between human vision and electronic devices. In addition, motivated by the visual simulation of photonic memristive and memristive‐like devices, various real human sensory characteristics, such as olfactory, ocular, and auditory, can be simulated by combining with touch sensors and gas sensors, which provides a new conception for realizing artificial robots.

Photonic memristive and memristive‐like devices have exhibited considerable promise in various applications even in its infancy. However, in the commercialization‐oriented approach, several difficulties, such as unclear mechanisms, diverse but not stable materials, and difficulty of integration, must be overcome. Here, we provide a commercialization‐oriented mini‐roadmap of photonic memristive and memristive‐like devices to guide the community's efforts:

The stability and external noise of the photonic memristive and memristive‐like devices are the primary variables impacting large‐scale applications as the fundamental original device for developing circuits. Starting from the lowest‐level device and improving the response noise and stability of a single device to light is the key to addressing this challenge, which involves the cross‐integration and growth of researchers from many domains. On a materials level, efforts should be made to interpret and effectively exert the light‐matter interaction between physical and chemical material properties using advanced technical means, as well as to regulate the photoresponse stably at the microscopic level. A general material design method is in urgent need to develop appropriate photo‐active materials for various application scenarios. On the hardware level, an optimization algorithm can be utilized to eliminate the performance drift of the device within the allowable range to meet the requirements of accurate calculation.

Currently, even though a large number of photonic memristive and memristive‐like devices have been proposed, research on them focuses primarily on the performance of a single device, whereas a general hardware architecture must be developed to realize a wider range of practical applications than just device performance improvement. Thereinto, the new hardware architecture requires co‐packaging thousands of photonic memristive and memristive‐like devices with peripheral electronic devices such as electronic controls, transistors, and especially lasers and amplifiers. Unfortunately, this is a huge challenge so far because co‐packaging the light source in photonic memristive and memristive‐like devices based‐chip is very difficult compared to a pure electrical case. More research efforts should be focused on the integration of light sources with photonic memristive and memristive‐like devices to develop a generic photonic chip. Another direction of effort is to develop specific small‐scale photonic memristive and memristive‐like devices integrated circuits for dedicated embedded, in which case the merits can be fully exploited without extremely high integration. Typically, these chips are designed for specific purposes, such as flexible electronic systems, tactile perception systems, and visual memory systems, thus a specific requirement in materials and structure of the photonic device is needed.

Another significant obstruction is the utilization of optical information. The speed, frequency, and bandwidth in optics are much larger than in electronics, which are critical for emulating the huge interconnectivity in the brain and bypassing and further constructing the efficient computing cores. However, the high‐speed transmission and processing of high‐bandwidth optical information in chips is a tough challenge to solve. A WDM technique has recently been proposed to solve the above problems. Although there has been a lot of study into using the unique qualities of WDM to provide high‐bandwidth and high‐frequency optical information transmission in phase‐change devices, it is not widely used in photonic memristive and memristive‐like devices. For better and more precise transmission and identification of optical information, more studies should be directed to the development of WDM technology.

In overview, as an emerging optoelectronic technology, photonic memristive and memristive‐like devices have attracted tremendous attention in the past few years due to their unique merits. In the future, creative and multidiscipline work, including chemistry, physics, material science, electrical engineering, computer science, and neuroscience, is needed to eventually apply photonic memristive and memristive‐like devices in novel memory and bionic electronics systems. Moreover, with a great number of continued efforts dedicated to the rapidly ongoing progress in photonic memristive and memristive‐like devices research, the high‐density memory and bionic chip based on photonic memristive and memristive‐like devices with high performances can be expected in years to come. Indeed, we firmly consider that this review provides a useful guide to facilitate the realization of this process and open up new ways for the further theoretical development of photonic memristive and memristive‐like devices.

## Conflict of Interest

The authors declare no conflict of interest.
